# Inactivation of EGLN3 hydroxylase facilitates Erk3 degradation via autophagy and impedes lung cancer growth

**DOI:** 10.1038/s41388-022-02203-2

**Published:** 2022-02-05

**Authors:** Ying Jin, Yamu Pan, Shuang Zheng, Yao Liu, Jie Xu, Yazhi Peng, Zemei Zhang, Yadong Wang, Yulian Xiong, Lei Xu, Kaiyu Mu, Suwen Chen, Fei Zheng, Ye Yuan, Jian Fu

**Affiliations:** 1 The Laboratory of Inflammation and Vascular Biology, Institute of Clinical Medicine and Department of Cardiology, Renmin Hospital, Hubei University of Medicine, Shiyan, Hubei China; 2 Aab Cardiovascular Research Institute and Department of Medicine, University of Rochester School of Medicine and Dentistry, Rochester, NY USA; 3 Department of Ultrasound, Renmin Hospital, Hubei University of Medicine, Shiyan, Hubei China; 4 Graduate School, Jinzhou Medical University, Jinzhou, Liaoning China

**Keywords:** Cancer microenvironment, Autophagy, Post-translational modifications

## Abstract

EGLN3 is critically important for growth of various cancers including lung cancer. However, virtually nothing is known about the role and mechanism for EGLN3 hydroxylase activity in cancers. EGLN3 catalyzes the hydroxylation of extracellular signal-regulated kinase 3 (Erk3), a potent driver of cancers. The role and mechanism for EGLN3-induced stabilization of Erk3 remain to be defined. Here, we show that Erk3 interacts with heat shock cognate protein of 70 kDa (HSC70) and lysosome-associated membrane protein type 2 A (LAMP2A), two core components of chaperone-mediated autophagy (CMA). As a consequence, Erk3 is degraded by the CMA-lysosome pathway. EGLN3-catalyzed hydroxylation antagonizes CMA-dependent destruction of Erk3. Mechanistically, hydroxylation blunts the interaction of Erk3 with LAMP2A, thereby blocking lysosomal decay of Erk3. EGLN3 inactivation inhibits macrophage migration, efferocytosis, and M2 polarization. Studies using EGLN3 catalytically inactive knock-in mice indicate that inactivation of EGLN3 hydroxylase in host cells ameliorates LLC cancer growth through reprogramming the tumor microenvironment (TME). Adoptive transfer of macrophages with inactivated EGLN3 restrains tumor growth by mounting anti-tumor immunity and restricting angiogenesis. Administration of EGLN3 hydroxylase pharmacologic inhibitor to mice bearing LLC carcinoma impedes cancer growth by targeting the TME. LLC cells harboring inactivated EGLN3 exhibit reduced tumor burden via mitigating immunosuppressive milieu and inducing cancer senescence. This study provides novel insights into the role of CMA in regulating Erk3 stability and the mechanism behind EGLN3-enhanced stability of Erk3. This work demonstrates that inactivation of EGLN3 in malignant and stromal cells suppresses tumor by orchestrating reciprocal interplays between cancer cells and the TME. This work sheds new light on the role and mechanism for EGLN3 catalytic activity in regulating cancer growth. Manipulating EGLN3 activity holds promise for cancer treatment.

## Introduction

Lung cancer underlies the leading cause of cancer-related morbidity and mortality worldwide [1]. Current regimens for lung cancer treatment largely focus on targeting cancer cells, which usually neglect the reciprocal interplays between malignant and stromal cells [2]. Macrophages are the most prevalent cell types within the tumor microenvironment (TME) [3]. Macrophages within the TME, designated tumor-associated macrophages (TAMs), drive tumor initiation, growth, metastasis, angiogenesis, efferocytosis, immunosuppression, and resistance to therapy [4]. Extensive macrophage infiltration correlates with poor prognosis of lung carcinoma [5]. Inhibiting macrophage recruitment into the TME was found to ameliorate tumor growth and development [6].

EGLN3 (also known as PHD3) belongs to the *Caenorhabditis elegans* gene egl-9 (EGLN) family of oxygen- and α-ketoglutarate-dependent prolyl hydroxylases [7]. EGLN3 is substantially inactivated under hypoxia in which EGLN3 is dramatically induced [7, 8]. Hypoxia is a hallmark of cancer [9]. There is a long-standing puzzle of the relevance for hypoxic induction of EGLN3 expression in cancers. EGLN3 catalyzes the hydroxylation of numerous proteins [7, 10–13]. EGLN3 exhibits a variety of biological functions, ranging from regulating cell signaling, cellular metabolism, cell cycle, apoptosis, and migration of cancer cells; and EGLN3 plays key roles in tumor growth and progression [13–19]. Notably, virtually all of these studies were carried out with cancer cells themselves. Although the significance of EGLN3 within cancer cells including lung cancer cells [17] has been extensively explored, the significance of EGLN3 hydroxylase activity in malignant and stromal cells remains poorly defined thus far. This study explored the role for EGLN3 hydroxylase activity in lung cancer growth.

Extracellular signal-regulated kinase 3 (Erk3, also known as MAPK6) is a newly identified substrate for EGLN3 hydroxylase [20]. The functional significance that EGLN3 stabilizes the Erk3 protein [20] remains to be defined. Erk3 expression was enhanced in lung carcinoma [21]. Erk3 is emerging as a potent driver that has been linked to the initiation, progression, and therapy of cancers including lung carcinoma [21–23]. However, the role of Erk3 in stromal cells (for instance, macrophages) is elusive. Another significant and open question is our poor understanding of the regulation of the Erk3 protein. Erk3 is a labile protein that undergoes decay through the ubiquitin (Ub)-proteasome pathway [24]. It remains to be determined whether cells could use the autophagy-lysosome mechanism to regulate Erk3 stability.

Our current study provides novel insights into the role of the chaperone-mediated autophagy (CMA) in Erk3 turnover and the mechanism for EGLN3-increased stability of Erk3. We showed that inactivation of EGLN3 in host cells retarded LLC lung cancer growth through programming the TME. Importantly, LLC lung cancer cells carrying inactive EGLN3 displayed a reduction of tumor burden via ameliorating immunosuppressive milieu and inducing LLC cell senescence. We demonstrate that inactivation of EGLN3 in malignant and stromal cells suppresses tumor by orchestrating the reciprocal interplays between cancer cells and the TME. Manipulating EGLN3 activity holds promise for the treatment of lung carcinoma.

## Results

### Genetic inactivation of EGLN3 hydroxylase inhibits Erk3 expression in mouse tissues and macrophages

**Fig. 1:**
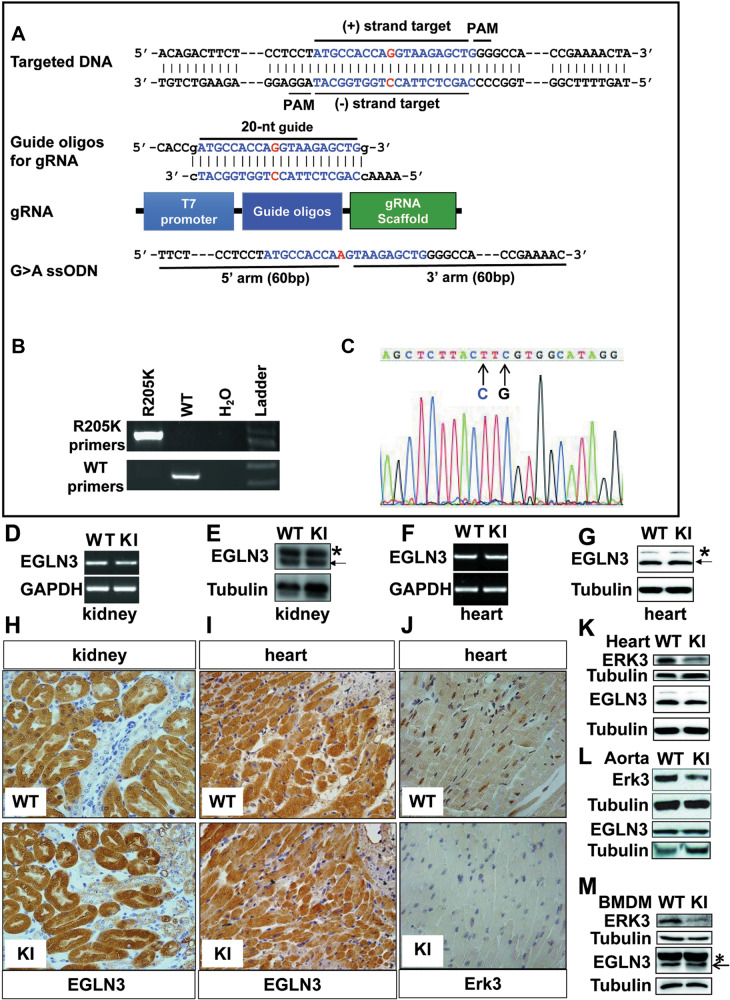
Generation and identification of mouse strain deficient for EGLN3 hydroxylase activity. **A** Shown is the strategy for the generation of EGLN3R205K knock-in (KI) mouse strain using CRISPR/Cas9 approach. **B** Genotyping of tail genomic DNA from wild-type or KI mouse by conventional PCR (*n* = 3). **C** DNA sequencing of genomic DNA from KI mouse (*n* = 2). **D**, **F** RT-PCR analysis of EGLN3 and R205K mRNA levels in the kidney (**D**) and heart (**F**) from wild-type or KI mouse (*n* = 3). **E**, **G** Immunoblotting analysis of EGLN3 and R205K protein levels in the kidney (**E**) and heart (**G**) from wild-type or KI mouse (*n* = 3). **H**, **I** Immunohistochemistry analysis of EGLN3 and R205K expression in the kidney (**H**) and heart (**I**) from wild-type or KI mouse (*n* = 3). **J** Immunohistochemistry analysis of Erk3 levels in the heart of wild-type or KI mouse (*n* = 3). **K**–**M** Immunoblotting analysis of Erk3 expression in the heart (**K**), aorta (**L**) and bone marrow-derived macrophages (BMDM) (**M**) of wild-type or KI mouse. GAPDH and Tubulin were used as the loading control (*n* = 3). CRISPR clustered regularly interspaced short palindromic repeats; ssODNs single-stranded DNA oligonucleotides; PAM protospacer adjacent motif; gRNA guide RNA; WT wild-type; KI knock-in; BMDM bone marrow-derived macrophages.

We and others have demonstrated that Arginine (R) 205 in mouse EGLN3 is absolutely required for its hydroxylase activity [25, 26]. To probe the biological and pathological importance of EGLN3 hydroxylase activity, we took advantage of CRISPR/Cas9 approach to generate a mouse strain deficient for EGLN3 hydroxylase activity by replacing R205 to lysine (K) (Fig. 1A). PCR and DNA sequencing analyses demonstrated that R205K mutation was successfully made in mouse EGLN3 (Fig. 1B, C). There are no overt changes in the development and fertility of R205K knock-in (KI) mice (data not shown). We would designate the mice KI mice for simplicity hereafter. Among the tissues examined, we found that EGLN3 was readily detected in the heart and kidney. The expression of R205K was similar to that of wild-type (WT) EGLN3 in the heart and kidney (Fig. 1, D–I).

To assess the EGLN3 hydroxylase activity in KI mice, we analyzed the expression of proteins that were reportedly hydroxylated and destabilized/stabilized by EGLN3, particularly those that play instrumental roles in cancers, such as Erk3, p53 and hypoxia-inducible factor (HIF) 1α [7, 20, 27]. Compared to that in the WT counterparts, Erk3 expression was substantially decreased in the heart (Fig. 1J, K), aorta (Fig. 1L) and mouse bone marrow-derived macrophages (BMDM) (Fig. 1M) of the KI mice. In contrast, there was minimal, if any, difference in the expression of HIF1α and p53 between the two genotypes (data not shown). Our observation that inactivation of EGLN3 did not alter HIF1α abundance is largely because EGLN1 rather than EGLN3 is critical for the regulation of HIF1α hydroxylation and degradation [28]. To clarify the effect of EGLN3 and its activity on p53 expression, we next conducted gain- and loss-of-function studies. Deletion of EGLN3 with its specific siRNA in LLC cells reduced p53 abundance (Supplementary Fig. 1A), while overexpression of R205K enhanced the expression of p53 to an extent similar to WT EGLN3 (Supplementary Fig. 1B). Notably, EGLN3 and R205K increased the protein rather than mRNA of p53 (Supplementary Fig. 1B). We also found that EGLN3 and R205K interacted with p53 (Supplementary Fig. 1C, D), which occurred primarily in the nuclei (Supplementary Fig. 1E). There was also no significant change in p53 abundance in KI BMDM compared to the WT counterpart (data not shown). Our data demonstrated that EGLN3 stabilized p53 independently of the hydroxylase activity, consistent with a recent finding [29]. Thus, Erk3 is a prominent substrate for EGLN3.

### EGLN3 protects against lysosomal degradation of Erk3

**Fig. 2:**
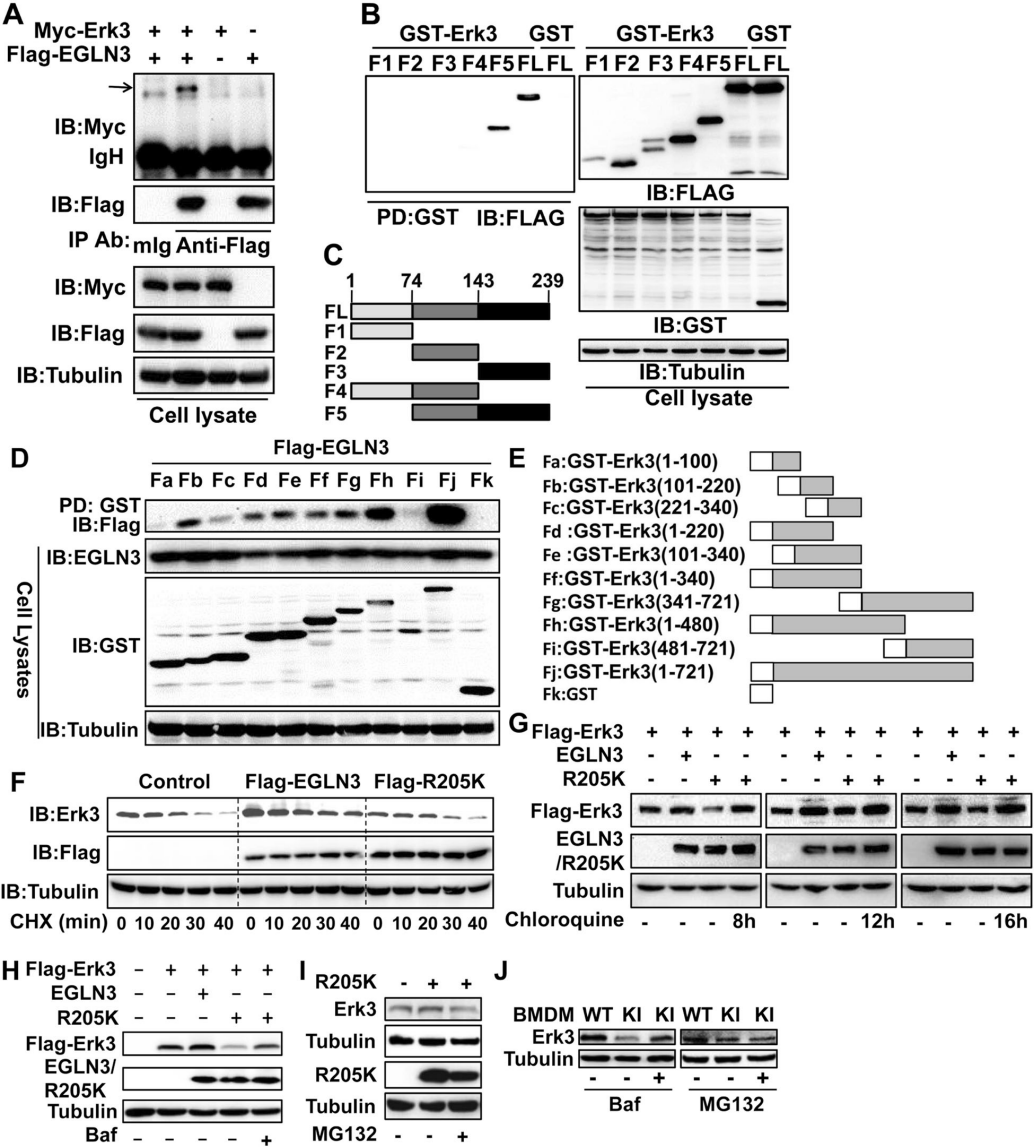
EGLN3 protects against lysosomal degradation of the Erk3 protein. **A** Co-immunoprecipitation (IP) and immunoblotting (IB) analysis of the Erk3-EGLN3 interaction. Total cell lysates prepared from HEK293T cells transfected with the indicated plasmids were immuoprecipitated with anti-Flag or control mouse IgG and then immunoblotted with anti-myc. **B** GST pulldown analysis of the interaction between Erk3 and EGLN3 or its fragments. Total cell lysates were incubated with Glutathione-Sepharose 4B beads and then immunoblotted with anti-Flag. **C** Schematic representation of full-length or different fragments of EGLN3. **D** GST pulldown analysis of the interaction between EGLN3 and Erk3 or its fragments. **E** Schematic representation of full-length or different fragments of Erk3. **F** Cycloheximide chase experiment was conducted to analyze the effect of EGLN3 or EGLN3R205K on Erk3 stability. HEK293T cells were transfected with the indicated plasmids and then treated with CHX (30 µg/mL) for the indicated times. Total cell lysates were analyzed by IB with the indicated antibodies. **G**–**J** HEK293T cells transfected with the indicated plasmids (**G**–**I**) or bone marrow-derived macrophages (**J**) were treated with Chloroquine (200 µM, 8, 12, and 16 h, respectively), Bafilomycin A1 (100 ng/mL, 16 h), or MG132 (10 µM, 8 h). IB was conducted with the indicated antibodies. GST glutathione S-transferase; IgH heavy chain of IgG; mIg mouse immunoglobulin; IP immunoprecipitation; IB immunoblotting; FL full-length; CHX cycloheximide; BMDM bone marrow-derived macrophages; PD pull-down; Ab antibody; Baf Bafilomycin A1; WT wild-type; KI knock-in.Shown are representative images of three independent experiments.

To uncover the mechanism whereby EGLN3 stabilizes the Erk3 protein, we began with analyzing the interaction between EGLN3 and Erk3. Co-immunoprecipitation (IP) and GST pulldown experiments indicated that EGLN3 interacted with Erk3 (Fig. 2A and Supplementary Fig. 2A), consistent with a previous study [20]. We then sought to map the binding region of each protein responsible for interaction with their binding partner. The region ranging from amino acid (aa) 74 through 239 was required for its interaction with Erk3 (Fig. 2B, C). Likewise, EGLN3 interacted with different regions of Erk3 with aa 340 through aa 480 being the prominent domain (Fig. 2D, E).

We next explored the functional significance of the EGLN3-Erk3 interaction. As expected, the expression of EGLN3 rather than R205K increased the level of the Erk3 protein (Supplementary Fig. 2B). Cycloheximide (CHX) chase experiment showed that expressing EGLN3 significantly dampened the decay of Erk3 (Fig. 2F and Supplementary Fig. 2C). In sharp contrast, R205K failed to do so (Fig. 2F). Taken together, Erk3-stabilizing effect of EGLN3 requires its hydroxylase activity.

The Ub-proteasome pathway was the only known mechanism regulating Erk3 stability to date [24]. However, we could not find reproducible and significant effect of EGLN3 on Erk3 ubiquitination (Supplementary Fig. 2D). Meanwhile, exposure of R205K-expressing cells to the proteasome inhibitor MG132 was unable to restore the Erk3 levels in HEK293T cells (Fig. 2I) and KI macrophages (Fig. 2J). Thus, it is unlikely that EGLN3 stabilizes Erk3 via the Ub-proteasome pathway. Instead, Erk3 expression in HEK293T cells can be efficiently rescued by chloroquine (Fig. 2G) and Bafilomycin A1 (Fig. 2H and Supplementary Fig. 2E), two well-known lysosome inhibitors [30, 31]. Bafilomycin A1 restored Erk3 expression in KI macrophages (Fig. 2J). Thus, EGLN3 protects against lysosomal degradation of the Erk3 protein.

### Erk3 is a novel substrate of the chaperone-mediated autophagy (CMA) pathway

**Fig. 3:**
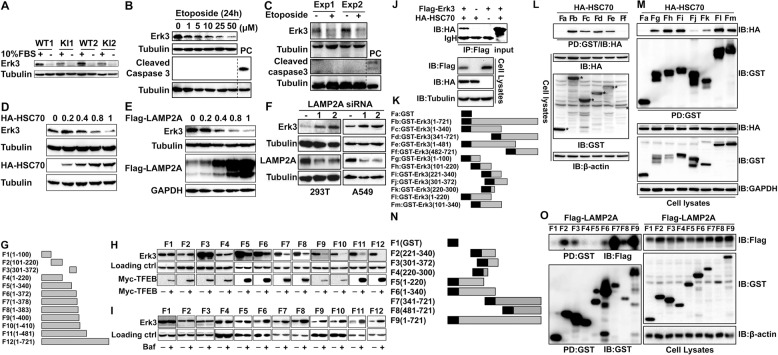
Erk3 is a novel substrate of the chaperone-mediated autophagy (CMA)-lysosome pathway. **A** Immunoblotting (IB) analysis of Erk3 expression in bone marrow-derived macrophages (BMDM) cultured in the presence (+) or absence (−) of 10% fetal bovine serum. **B** IB analysis of cell lysates prepared from BMDM treated with different concentrations of etoposide for 24 h. **C** IB analysis of total cell lysates from the mouse hearts treated with etoposide (50 mg/kg body weight, 24 h). **D**, **E** HEK293T cells were transfected different amounts (0–1 µg) of HA-HSC70 or Flag-LAMP2A. Cell lysates were immunoblotted as indicated. **F** HEK293T and A549 cells were transfected with control siRNA (−) or two sets of Lamp2A siRNAs. Total cell lysates were analyzed for the expression of proteins indicated. **G** Schematic representation of full-length or different fragments of Erk3 used for the experiments depicted in panels (**H**) and (**I**). **H** IB analysis of cell lysates from HEK293T cells transfected with the indicated plasmids. **I** IB analysis of cell lysates prepared from HEK293T cells transfected with the indicated plasmids in the presence of Bafilomycin A1 (100 ng/mL, 16 h). **J** Total cell lysates from HEK293T cells transfected as indicated were immunoprecipitated by anti-Flag and then immunoblotted with anti-HA. **K** Schematic representation of full-length or different fragments of Erk3 used for the experiments presented in panels (**L)** and (**M**). **L**, **M** GST pulldown analysis of the interaction between HSC70 and full-length or different fragments of Erk3. **N** Schematic representation of full-length or different fragments of Erk3 used for experiments depicted in panel (**O**). **O** GST pulldown analysis of the interaction between LAMP2A and full-length or different fragments of Erk3. BMDM bone marrow-derived macrophages; FBS fetal bovine serum; WT wild-type; KI knock-in; PC positive control; HSC70 heat shock cognate protein of 70 kDa; LAMP2A lysosome-associated membrane protein type 2A; exp experiment; ctrl. control; IgH heavy chain of IgG; IP immunoprecipitation; IB immunoblotting; PD pull down; GST glutathione S-transferase; siRNA small interfering RNA. Shown are representative images of three independent experiments.

Serum deprivation significantly decreased Erk3 expression in various types of cells examined, including primary macrophages (Fig. 3A), HEK293T cells (Supplementary Fig. 3A), and liver cancer Hep3B cells (Supplementary Fig. 3B). The Erk3 protein level was also markedly reduced by the DNA-damage reagent etoposide in macrophages (Fig. 3B) and HEK293T cells (Supplementary Fig. 3C) in a dose- and time-dependent manners (Supplementary Fig. 3C). Furthermore, mouse studies showed that administration of etoposide pronouncedly suppressed Erk3 expression in the heart (Fig. 3C). Notably, the inhibitory effect of etoposide was not due to its apoptosis-inducing role (Fig. 3B, C). Our observations suggested that the CMA might be used to target Erk3 for lysosomal destruction.

The cytosolic heat shock cognate protein of 70 kDa (HSC70, also known as HSPA8) contributes to the recognition and transport of the CMA substrate [32]. Meanwhile, the lysosome-associated membrane protein type 2 A (LAMP2A) is responsible for binding and then delivering the substrate protein into the lysosomal lumen for destruction [32]. We thus evaluated the effect of both molecules on Erk3 abundance. Expressing HSC70 repressed the abundance of endogenous and exogenous Erk3 in a concentration–dependent fashion (Fig. 3D and Supplementary Fig. 3D). Importantly, expressing LAMP2A reduced the abundance of Erk3 (Fig. 3E and Supplementary Fig. 3E). Considering LAMP2A is an essential CMA component, we further interrogated its role in regulating Erk3 expression by conducting loss-of-function studies with two sets of LAMP2A siRNAs. Both pairs of LAMP2A siRNAs were capable of reducing the expression of endogenous LAMP2A (Fig. 3F and Supplementary Fig. 3G). Impressively, deletion of LAMP2A markedly enhanced the expression of the Erk3 protein in both HEK293T and A549 cells (Fig. 3F). Accordingly, LAMP2A negatively regulates Erk3 expression.

We then provided evidence for the implication of lysosomes in regulating Erk3 abundance. The transcription factor EB (TFEB) is a master regulator of lysosomal biogenesis that is required for autophagy [33]. Expressing TFEB dramatically reduced Erk3 levels (Supplementary Fig. 3F), indicating that Erk3 can be degraded by lysosomes. To verify and extend these findings, we prepared serious constructs expressing differential regions of Erk3 (Fig. 3G). TFEB markedly decreased the expression of a fragment from aa 1 to 340 or greater ones (Fig. 3H). As expected, the lysosome inhibitor Bafilomycin A1 executed an opposite role (Fig. 3I). Collectively, Erk3 is subjected to lysosomal proteolysis through the CMA-lysosome pathway.

### Erk3 physically interacts with HSC70 and LAMP2A

We next examined whether HSC70 and LAMP2A physically interact with Erk3. Co-IP and GST pulldown experiments revealed an interaction between HSC70 and Erk3 (Fig. 3J, L). We then mapped the region within Erk3 critical for HSC70 binding with a set of constructs expressing differential regions of Erk3 (Fig. 3K; Supplementary Fig. 4, A-I). Although HSC70 bound different regions of Erk3, the N terminal 300 amino acids of Erk3 play a dominant role in mediating such an interaction (Fig. 3L, M; Supplementary Fig. 4, A-I). Likewise, LAMP2A is a novel binding protein for Erk3 (Supplementary Fig. 4J). We further confirmed and extended this finding with constructs expressing diverse parts of Erk3 (Fig. 3N; Supplementary Fig. 4K, L). Several regions within the Erk3 contribute to LAMP2A binding with the one harboring aa 300–480 predominating LAMP2A binding (Fig. 3O; Supplementary Fig. 4K, L). Importantly, HSC70 and LAMP2A did not compete with each other for Erk3 binding (Supplementary Fig. 4M, N), indicating that HSC70 and LAMP2A binds different regions of the Erk3 protein. Taken together, Erk3 physically and functionally interacts with HSC70 and LAMP2A.

### EGLN3 antagonizes the interaction of Erk3 with HSC70 and LAMP2A

**Fig. 4:**
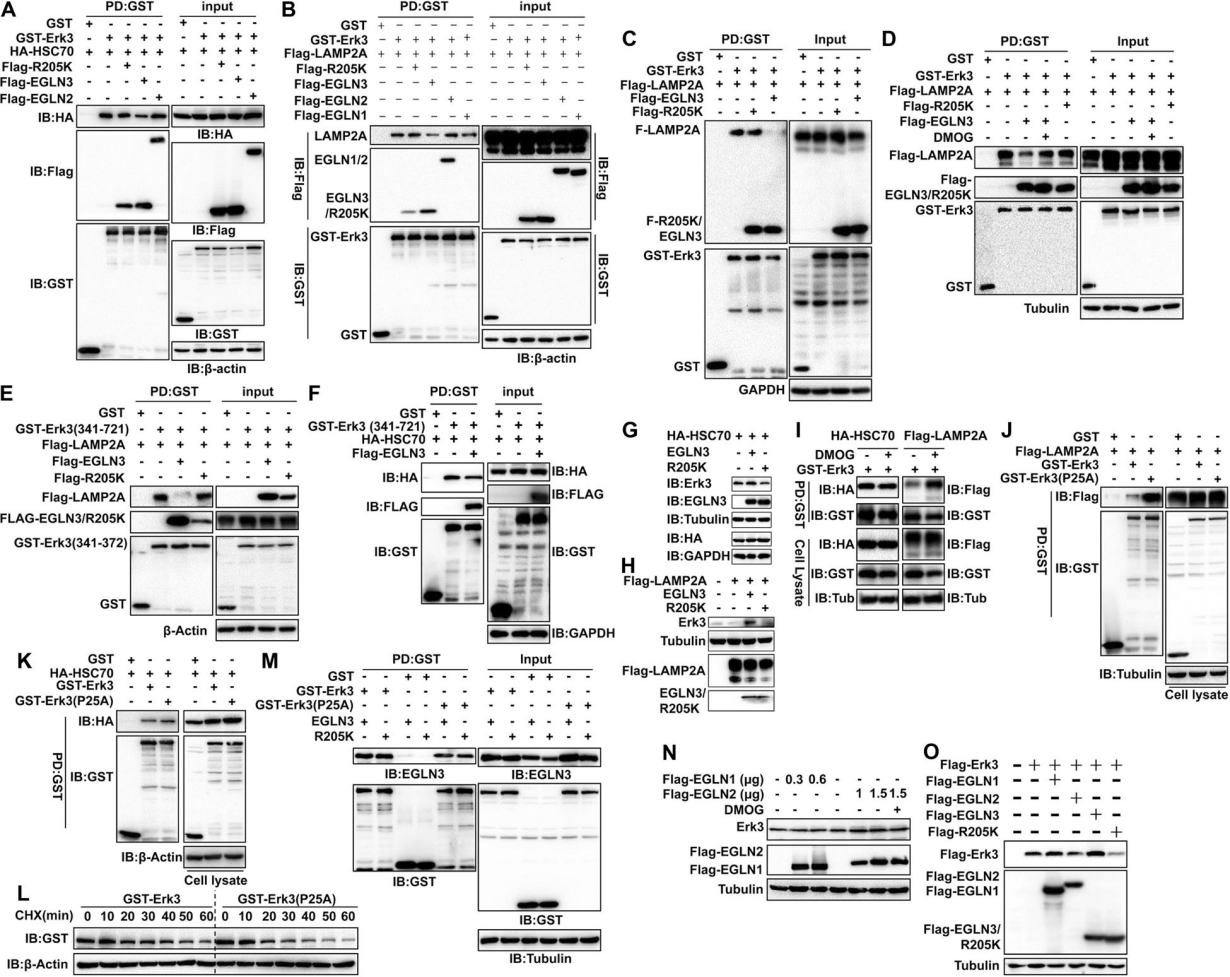
EGLN3 selectively antagonized Erk3 interaction with HSC70 and LAMP2A in a catalytic activity-dependent manner. **A** GST pulldown analysis of the effect of EGLN2, EGLN3, or R205K on the interaction between Erk3 and HSC70 (*n* = 3). **B** GST pulldown analysis of the effect of EGLN1, EGLN2, EGLN3, or R205K on the interaction between Erk3 and LAMP2A (*n* = 3). **C** GST pulldown analysis of the impact of EGLN3 and R205K on the interaction between Erk3 and LAMP2A (*n* = 3). **D** GST pulldown analysis of the impact of EGLN3 activity on the interaction between Erk3 and LAMP2A (*n* = 4). **E**, **F** GST pulldown analysis of the effect of EGLN3 on Erk3(341–721) interaction with LAMP2A (**E**) and HSC70 (**F**) (*n* = 3). **G**, **H** IB analysis of the influence of EGLN3 and R205K on Erk3 degradation induced by HSC70 or LAMP2A (*n* = 3). **I** IB analysis of the effect of DMOG on Erk3 interaction with HSC70 or LAMP2A (*n* = 3). **J**, **K** GST pulldown analysis of Erk3 or hydroxylation-resistant mutant Erk3(P25A) interaction with LAMP2A (**J**) and HSC70 (**K**) (*n* = 3). **L** Cycloheximide chase experiment was conducted to measure the stability of Erk3 or its mutant Erk3(P25A) (*n* = 3). **M** GST pulldown analysis of Erk3 or hydroxylation-resistant mutant Erk3(P25A) interaction with EGLN3 or R205K (*n* = 3). **N**, **O** IB analysis was done to examine the expression of relative proteins in HEK293T cells transfected the indicated constructs (*n* = 3). Tub tubulin; HSC70 heat shock cognate protein of 70 kDa; LAMP2A lysosome-associated membrane protein type 2A; PD pull down; GST glutathione S-transferase; IB immunoblotting; DMOG dimethyl oxalyglycine; CHX cycloheximide.

Physical interaction with HSC70 and LAMP2A is a prerequisite for a substrate to be degraded by the CMA-lysosome pathway [32, 34]. We wondered whether EGLN3 interferes with the Erk3-HSC70 and/or Erk3-LAMP2A interactions. Interestingly, the interaction between HSC70 and Erk3 was counteracted by EGLN3 (Fig. 4A and Supplementary Fig. 5A). Likewise, the interaction of Erk3 with LAMP2A was also compromised by EGLN3 (Fig. 4, B–D; Supplementary Fig. 5B). EGLN3 substantially reduced the Erk3(1–340)-HSC70 and Erk3(1–340)-LAMP2A interactions (Supplementary Fig. 5C, D). While EGLN3 robustly interrupted the Erk3(341–721)-LAMP2A interaction (Fig. 4E), it barely blunted the Erk3(341–721)-HSC70 interaction (Fig. 4F). These observations are consistent with our domain-mapping results. In sum, EGLN3 antagonizes the interaction of Erk3 and LAMP2A and HSC70.

### Inactivation of EGLN3 activity impairs the ability of EGLN3 to counteract the interaction of Erk3 with HSC70 and LAMP2A

We next determined whether hydroxylase activity is essential for the ability of EGLN3 to impede the Erk3-HSC70 and Erk3-LAMP2A interactions. As anticipated, Erk3 interacted with HSC70 and with LAMP2A, respectively (Fig. 4A, B; Supplementary Fig. 5E, F). EGLN3, but not R205K, was able to hinder the Erk3-HSC70 (Fig. 4A) and Erk3-LAMP2A (Fig. 4B, C) interactions. Notably, GST pulldown and co-IP experiments showed that there was no significant difference in the Erk3-EGLN3 or Erk3-R205K interaction (Fig. 4M and Supplementary Fig. 5G). This finding excluded the likelihood that competition with HSC70 or LAMP2A for Erk3 binding accounts for EGLN3 blockade of the Erk3-HSC70 and Erk3-LAMP2A interactions. Impressively, the effect of EGLN3 on the Erk3-LAMP2A interaction was abrogated by DMOG, a well-recognized pharmacologic inhibitor for EGLN3 hydroxylase (Fig. 4D). Taken together, inhibition of EGLN3 hydroxylase activity primarily impaired the ability of EGLN3 to restrain the association of Erk3 with LAMP2A.

Consistent with their role in regulating the physical interaction of proteins, EGLN3 rather than R205K abolished the Erk3-destabilizing effect of HSC70 (Fig. 4G) and LAMP2A (Fig. 4H). Thus, EGLN3 antagonizes the physical and functional interactions of Erk3 with LAMP2A and HSC70, which is dependent on its hydroxylase activity.

### Hydroxylation stabilizes Erk3 by blocking its interaction with LAMP2A

We tested whether EGLN3 stabilizes Erk3 through catalyzing the hydroxylation of Erk3 using EGLN3 inhibitor DMOG and Erk3 hydroxylation-resistant mutant Erk3P25A [20]. Fig 4I showed that DMOG dramatically increased the Erk3-LAMP2A but not Erk3-HSC70 interaction. Furthermore, Erk3P25A displayed much stronger ability than Erk3 to bind LAMP2A (Fig. 4J), while there was no significant difference in HSC70 interaction with Erk3 or with Erk3P25A (Fig. 4K). CHX chase experiment indicated that the stability of the Erk3P25A protein was substantially declined compared with that of the Erk3 (Fig. 4L and Supplementary Fig. 6). Intriguingly, Erk3P25A retained the ability to bind EGLN3 and R205K (Fig. 4M and Supplementary Fig. 5G). Thus, hydroxylation blunts Erk3 interaction with LAMP2A and thereby protects against Erk3 destruction by the CMA. In summary, EGLN3 stabilizes Erk3 primarily through promoting its hydroxylation and thereby preventing Erk3 from its degradation by the CMA pathway.

### EGLN3 selectively stabilizes the Erk3 protein

There are three members in the EGLN family of hydroxylases in mammalian cells, namely EGLN1, 2, and 3. We determined whether they all are capable of stabilizing Erk3. As a first step, we explored whether Erk3 interacted with EGLN1 and 2. We found that Erk3 interacted with EGLN2 and EGLN3, but not EGLN1 (Supplementary Fig. 7A, B). Unlike EGLN3, EGLN2 failed to inhibit the interaction of Erk3 with HSC70 and with LAMP2A (Fig. 4A, B). Additionally, EGLN1 was inefficacious in regulating the Erk3-LAMP2A (Fig. 4B) and Erk3-HSC70 (data not shown) interactions. As expected, EGLN1 and 2 had no discernible impact on Erk3 expression (Fig. 4N, O). Thus, EGLN3 selectively protects against lysosomal degradation of Erk3.

### Erk3 promotes macrophage migration and neuropilin 1 expression

**Fig. 5:**
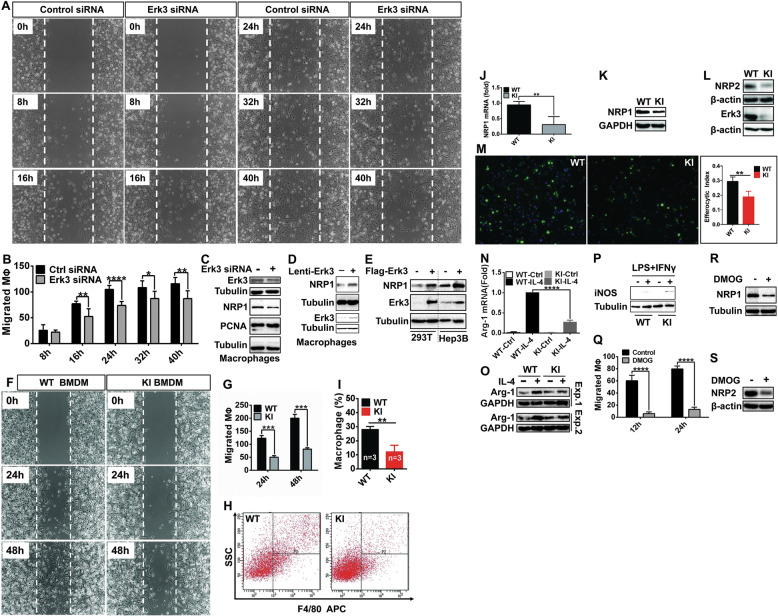
The effects of Erk3 and EGLN3 inactivation on gene expression and properties of macrophages. **A**, **B** Wound assay was conducted to determine the role for Erk3 in macrophage migration. BMDM were transfected with control or Erk3 siRNA for 48 h and then scratched. The cells in the denuded regions were counted and statistically analyzed. **C** IB analysis of the expression of proteins indicated in BMDM treated with control (−) or Erk3 (+) siRNA. **D**, **E** IB analysis of NRP1 expression in macrophages infected with lentivirus expressing Erk3 (**D**) or HEK293T or Hep3B cells transfected with plasmid expressing Flag-Erk3 (**E**). **F**, **G** Wound assay to evaluate the role for EGLN3 inactivation in macrophage migration. **H**, **I** in vivo migration of macrophages. Peritoneal macrophages in the lavages were collected from WT and KI mice injected intraperitoneally with thioglycollate, stained with PE-conjugated anti–F4/80 and quantified by flow cytometry. **J** qPCR analysis of NRP1 mRNA expression in WT and KI BMDM. **K**, **L** IB analysis of NRP1 and NRP2 expression in WT and KI BMDM. **M** Efferocytosis assay was performed to evaluate the ability of WT and KI BMDM to take up apoptotic Jurkat T cells labeled with Calcein AM. Efferocytosis was quantitated. **N**, **O** The expression of Arg1 mRNA (**N**) and protein (**O**) in WT and KI BMDM treated with (+) or without (−) IL-4 were analyzed by qPCR and IB analysis, respectively. **P** IB analysis of iNOS expression in WT and KI BMDM treated with (+) or without (−) LPS and IFNγ. **Q** Wound assay was pursued to evaluate the effect of DMOG on macrophage migration. **R**, **S** IB analysis of NRP1 and NRP2 expression in DMOG-treated BMDM. BMDM bone marrow-derived macrophages; siRNA small interfering RNA; NRP neuropilin; IB immunoblotting; DMOG dimethyl oxalyglycine; WT wild-type; KI knock-in; Arg1 arginase 1; iNOS inducible nitric oxide synthase; IL-4 interleukin 4; LPS lipopolysaccride; IFNγ interferon γ; *p < 0.05; **p < 0.01; ***p < 0.001; ****p < 0.0001 by unpaired 2-tailed Student’s *t* test. Shown are representative results of three independent experiments.

Having unraveled the mechanism that EGLN3 inactivation destabilizes Erk3, we clarified the biological consequence of this alteration in macrophages. Deletion of Erk3 by its specific siRNA pronouncedly impeded migration of macrophages into the denuded region (Fig. 5A, B), as demonstrated by wound healing assay. Similar results were achieved with another Erk3 siRNA (data not shown). However, Erk3 inhibition had no significant impact on the expression of PCNA, a marker for cell proliferation (Fig. 5C).

Loss of neuropilin (NRP) 1 on macrophages inhibited their recruitment into tumors and blunted the pro-angiogenic and immunosuppressive potential of TAMs [6]. Knockdown of Erk3 declined NRP1 expression (Fig. 5C), while forced expression of Erk3 conducted an opposite effect in macrophages (Fig. 5D), HEK293T cells and Hep3B cells (Fig. 5E). Accordingly, Erk3 enhances NRP1 expression in different types of cells examined.

### Genetic inactivation of EGLN3 hydroxylase inhibits macrophage migration, efferocytosis, and M2 polarization

Since Erk3 expression was reduced in KI macrophages, we tested the impact of EGLN3 inactivation on macrophage migration. In vitro migration of KI macrophages was strongly retarded (Fig. 5F, G). We then assessed in vivo migratory capacity of KI macrophages. The ability of macrophages to migrate was substantially impaired in KI mice compared to that in WT mice (Fig. 5H, I). EGLN3 inactivation reduced the expression of both NRP1 mRNA (Fig. 5J) and protein (Fig. 5K). However, EGLN3 inactivation had minimal, if any, effect, on the expression of MMP2, a matrix metalloproteinase associated with macrophage migration (Supplementary Fig. 8A).

We next addressed whether inactivation of EGLN3 also influences other properties of macrophages. There was no significant difference in apoptosis and proliferation between WT and KI macrophages (Supplementary Fig. 8B, C). In addition, WT and KI macrophages displayed no significant difference in NFкB signaling activation in response to LPS (Supplementary Fig. 8D).

The ability of macrophages to migrate toward apoptotic cells is important for efferocytosis, a process by which phagocytes (primarily macrophages) engulf apoptotic cells [35]. Excessive efferocytosis is immunosuppressive [35]. Ablation of macrophage NRP2 was known to impair efferocytosis and reinitiate antitumor adaptive immune response [36]. Fig 5L showed that EGLN3 inactivation reduced NRP2 expression. As shown in Fig. 5M, KI macrophages exhibited a reduced ability to take up apoptotic Jurkat T lymphocytes. Thus, EGLN3 inactivation inhibits efferocytosis of macrophages.

Efferocytosis reprograms macrophages toward an M2 phenotype [36]. As expected, EGLN3 inactivation attenuated interleukin 4-induced M2 polarization, as indicated by decreased mRNA and protein expression of arginase (Arg) 1 (Fig. 5N, O), a marker of M2 macrophages [37]. Albeit to a less extent, EGLN3 inactivation promoted M1 polarization of macrophages, as judged by the expression of inducible nitric oxide synthase (iNOS) (Fig. 5P), a hallmark of M1 macrophages [38]. Taken together, EGLN3 inactivation reduces the expression of NRP1 and NRP2. Inactivation of EGLN3 in macrophages inhibits their migration, efferocytosis and M2 polarization.

### Pharmacologic inhibition of EGLN3 hydroxylase retards macrophage migration

We next took advantage of pharmacologic means to confirm the major findings achieved with KI macrophages. Exposure of macrophages to DMOG potently attenuated macrophage migration (Fig. 5Q and Supplementary Fig. 9A). DMOG markedly reduced the expression of NRP1 (Fig. 5R) and NRP2 (Fig. 5S). Neither proliferation nor apoptosis were affected by DMOG (Supplementary Fig. 9, B-D). Collectively, pharmacologic inhibition of EGLN3 hydroxylase recapitulated the findings achieved by genetic approach.

### Inactivation of host EGLN3 hydroxylase activity restricted tumor growth of LLC lung carcinoma through mounting anti-tumor immunity and repressing angiogenesis

**Fig. 6:**
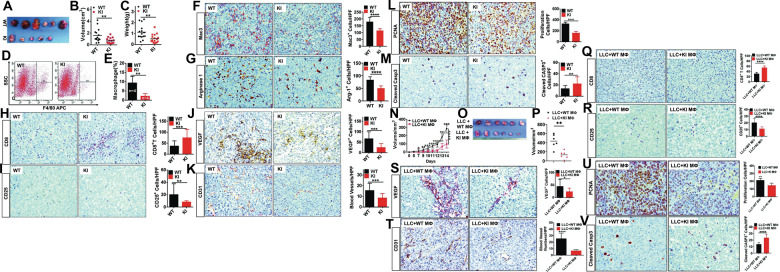
The role and mechanism of EGLN3 inactivation in host cells (particularly macrophages) in suppressing LLC tumor growth. **A**–**C** Tumor xenograft experiments were conducted by subcutaneous inoculation of LLC lung cancer cells into WT and KI mice. LLC tumors were dissected on day 21 after implantation. Tumor width, length and mass were measured. Tumor volumes were calculated. Shown are representative photograph of LLC tumors (**A**), tumor volume (**B**, *n* = 15 for WT and *n* = 17 for KI) and tumor mass (**C**, *n* = 15 for WT and *n* = 17 for KI). **D**, **E** Flow cytometry analysis of the content of macrophages prepared from WT and KI mice (*n* = 4). **F**–**M** Immunohistochemistry (IHC) analysis and quantification of macrophage content (**F**), arginase 1^+^ cells (**G**), CD8^+^ cells (**H**), CD25^+^ cells (**I**), VEGF expression (**J**), CD31^+^ cells (**K**), PCNA expression (**L**), and cleaved caspase 3 expression (**M**) in LLC tumors. **N**–**P** BMDM from WT (WT Mø) and KI (KI Mø) were subcutaneously injected into mice together with LLC cancer cells. LLC tumors were dissected on day 14 after implantation. Shown are tumor growth rate (**N**), representative photograph of LLC tumors (**O**), and tumor volume (**P**) (*n* = 6). **Q**–**V** IHC analysis and quantification of CD8^+^ cells (**Q**), CD25^+^ cells (**R**), VEGF expression (**S**), CD31^+^ cells (**T**), PCNA expression (**U**), and cleaved caspase 3 expression (**V**) in LLC tumors. HPF high power field; WT wild-type; KI knock-in; IHC immunohistochemistry; Casp3 Caspase 3; *p < 0.05; **p < 0.01; ***p < 0.001; ****p < 0.0001 by unpaired 2-tailed Student’s *t* test.

We tested the surmise that EGLN3 inactivation in host cells is protective against tumor growth. To this end, WT and KI mice were challenged with LLC lung carcinoma cells. Compared with WT mice, KI mice exhibited a small tumor burden, as judged by reduced tumor size and weight (Fig. 6, A–C). The LLC tumors in KI mice had much less macrophage infiltration than those in WT mice, as demonstrated by flow cytometry analysis (Fig. 6D, E) and immunohistochemistry (IHC) staining (Fig. 6F). More importantly, the content of Arg1 positive macrophages in tumors in KI mice was much less than that in WT mice (Fig. 6G). Clearly, inactivation of host EGLN3 hydroxylase reduced the content of M2 TAMs. M2 macrophages restrict the recruitment of tumor-protective CD8^+^ T lymphocytes while promoting the expansion of tumor-permissive regulatory T (Treg) cells [39]. As anticipated, EGLN3 inactivation increased in CD8^+^ T recruitment (Fig. 6H) but decreased Treg infiltration (Fig. 6I) in tumors. TAMs drive tumor growth and progression through diverse mechanisms involving promotion of immune suppression and angiogenesis [4, 40]. We then determined the impact of EGLN3 inactivation on tumor angiogenesis. Tumors from KI mice had much less expression of vascular endothelial growth factor (VEGF), the most important angiogenic factor, than those from WT mice (Fig. 6J). The micro-vessel density, as monitored by the expression of endothelial marker CD31, in tumors from KI mice was markedly lower than that in tumor from WT mice (Fig. 6K). Thus, the inactivation of EGLN3 hydroxylase in host cells inhibited tumor angiogenesis. EGLN3 inactivation suppressed proliferation (Fig. 6L) and potentiated apoptosis (Fig. 6M) of LLC cells.

### Macrophages with inactive EGLN3 restrained tumor growth

To provide direct evidence for cancer-protective role of EGLN3 hydroxylase-deficient macrophages, we co-injected WT and KI macrophages into WT mice with LLC cancer cells. Tumor growth in mice received KI macrophages was significantly restrained compared to that in mice received WT macrophages (Fig. 6, N to P). These data indicated that macrophages with inactive EGLN3 display tumor-suppressive potential. The content of CD8^+^ T cells in LLC tumors was pronouncedly increased in mice transferred KI macrophages when compared with that in mice received WT macrophages (Fig. 6Q). Adoptive transfer of KI macrophages dramatically reduced Treg infiltration (Fig. 6R). Importantly, the transfer of KI macrophages gave rise to a decline in VEGF expression (Fig. 6S) and micro-vessel density (Fig. 6T) in comparison to the receipt of WT macrophages. Intriguingly, the treatment of mice with KI macrophages led to decreased proliferation (Fig. 6U) and increased apoptosis (Fig. 6V) of LLC cells. Collectively, macrophages deficient for EGLN3 hydroxylase activity are tumor protective.

### Pharmacologic inhibitor of EGLN3 has potential to ameliorate tumor growth

**Fig. 7:**
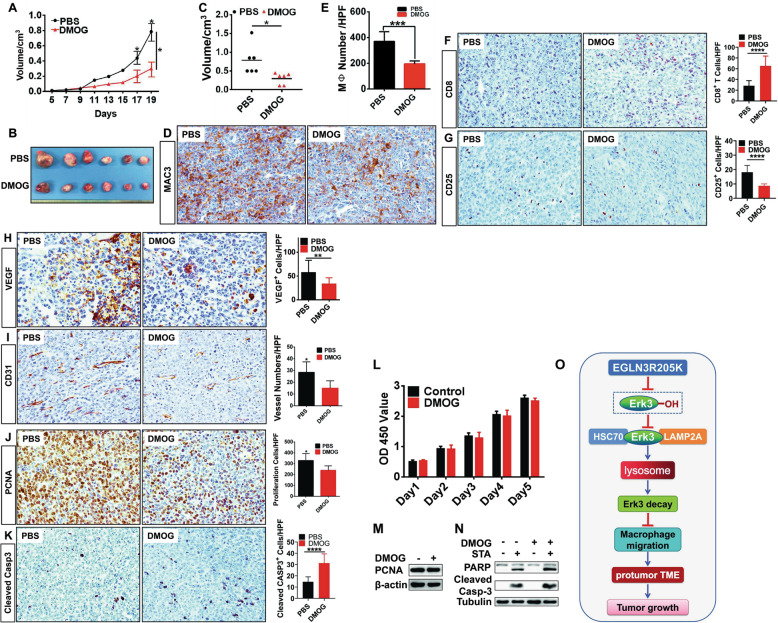
Administration of DMOG into mice bearing LLC lung cancer cells impeded tumor growth. LLC cancer cells were subcutaneously inoculated into mice. Mice bearing LLC tumors were daily administrated PBS or DMOG (200 mg/kg body weight) intraperitoneally at day 6 of tumor implantation. LLC tumors were dissected on day 19 after inoculation. Shown are tumor growth rate (**A**), representative photograph of LLC tumors (**B**), and tumor volume (**C**) (*n* = 6); **D**–**K** Immunohistochemistry analysis and quantification of macrophage content (**D**, **E**), CD8^+^ cells (**F**), CD25^+^ cells (**G**), VEGF expression (**H**), CD31^+^ cells (**I**), PCNA expression (**J**), and cleaved caspase 3 expression (**K**) in LLC tumors. **L** CCK8 assay was performed to estimate the influence of DMOG on the proliferation of LLC lung cancer cells (*n* = 3). **M** IB was conducted to monitor the expression of PCNA in LLC lung cancer cells treated with DMOG ( + ) or PBS (−) (*n* = 3). **N** IB was carried out to measure the expression of PARP and cleaved caspase 3 in LLC lung cancer cells exposed to STA or DMOG (*n* = 3). **O** Working model of genetic and pharmacologic inactivation of EGLN3 hydroxylase activity. HPF high power field; DMOG dimethyl oxalyglycine; STA staurosporine; IB immunoblotting; Casp3 Caspase 3; *p < 0.05; **p < 0.01; ***p < 0.001; ****p < 0.0001 by unpaired 2-tailed Student’s *t* test.

The selective pharmacologic inhibitors for EGLN3 are currently being developed. Herein, we performed a preclinical proof-of-principle study using DMOG to treat mice bearing LLC tumor. Administration of DMOG significantly ameliorated tumor growth in mice (Fig. 7, A–C). Treatment of LLC-bearing mice with DMOG substantially repressed macrophage infiltration (Fig. 7D, E), increased CD8^+^ T content (Fig. 7F), and reduced Treg abundance (Fig. 7G). Additionally, treatment of LLC-bearing mice with DMOG reduced VEGF expression (Fig. 7H) and micro-vessel density (Fig. 7I). Treatment of mice with DMOG attenuated proliferation (Fig. 7J) and potentiated apoptosis (Fig. 7K) of cancer cells. Notably, DMOG showed no significant effect on proliferation (Fig. 7L, M) and apoptosis (Fig. 7N) of cultured LLC cells in vitro. Thus, DMOG treatment mitigated cancer growth likely through reshaping the TME. Our findings support the rationale for using the EGLN3 hydroxylase inhibitor to treat lung cancer.

Collectively, our studies provided strong evidence that inactivation of EGLN3 hydroxylase activity in stromal cells could significantly blunt tumor growth through orchestrating the cross-talk between cancer cells and the TME (Fig. 7O).

### Expression of hydroxylase-inactive EGLN3 in LLC lung cancer cells impeded tumor growth by enhancing senescence of LLC cells

**Fig. 8:**
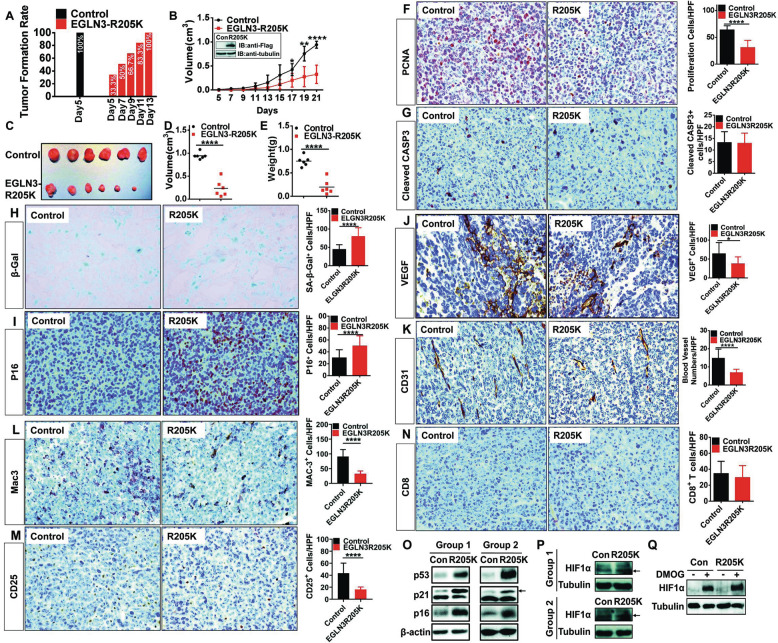
LLC lung cancer cells harboring hydroxylase-inactive EGLN3 exhibited reduced tumor growth. **A**–**E** LLC stable cell line expressing hydroxylase-inactive R205K or harboring control vector (**B**, inset) were subcutaneously inoculated into mice. LLC tumors were dissected on day 21 after implantation. Shown are tumor formation rate (**A**), tumor growth rate (**B**), representative photograph of LLC tumors (**C**), tumor volume (**D**) and tumor mass (**E**) (*n* = 6). **F**, **G** Immunohistochemistry analysis and quantification of PCNA expression (**F**) and cleaved caspase 3 expression (**G**) in tumors. **H** β-galactosidase staining and quantification of senescence-associated β-galactosidase (SA-β-Gal). **I**–**N** Immunohistochemistry analysis and quantification of p16 expression (**I**), VEGF expression (**J**), CD31^+^ cells (**K**), macrophage content (**L**), CD25^+^ cells (**M**), and CD8^+^ cells (**N**) in tumors. **O**, **P** IB analysis of tumor proteins prepared from the control and R205K tumors for the expression of proteins (p53, p21, p16, and HIF1α). **Q** IB analysis of HIF1α expression in control and R205K LLC cells (*n* = 3). Arrow indicates the specific band. HPF high power field; IB immunoblotting; Casp3 Caspase 3; β-Gal β-galactosidase; SA-β-Gal senescence-associated β-galactosidase; HIF1α hypoxia-inducible factor 1α; *p < 0.05; **p < 0.01; ****p < 0.0001 by unpaired 2-tailed Student’s *t* test.

EGLN3 hydroxylase is substantially inactivated but greatly induced under hypoxia, a hallmark of cancers [7, 9]. The role for hypoxic induction of EGLN3 expression is unknown. We would next investigate the significance of inactivated EGLN3 hydroxylase in LLC cancer cells in tumor growth. WT mice were implanted with LLC cell line stably expressing moderate levels of inactivated EGLN3 (R205K) or control LLC cells (Fig. 8B, inset). All mice developed palpable tumors at day 5 post-inoculation of the control LLC cells, when did only 33.3% of mice injected R205K-expressing LLC cells have tumor burden (Fig. 8A). All of mice received R205K-expressing LLC had palpable tumors as late as day 13 after inoculation (Fig. 8A). Mice carrying R205K-expressing LLC cells exhibited a reduction of tumor burden compared with those with control LLC cells (Fig. 8, B–E). Therefore, inactivated EGLN3 in LLC cells exhibited a tumor-inhibitory effect. Proliferation of R205K-expressing LLC cells in mice was profoundly impeded compared to that of control LLC cells (Fig. 8F). Apoptosis of R205K-expressing LLC cells occurred to an extent similar to that of control LLC cells (Fig. 8G). However, expressing R205K did not affect in vitro cell apoptosis (Supplementary Fig. 10A, B) and proliferation (Supplementary Fig. 10C).

Cellular senescence is a well-known cell-intrinsic mechanism to prevent tumorigenesis [41]. R205K-expressing LLC tumors exhibited an increased senescence, as judged by an increased lysosomal senescence-associated-β-galactosidase (SA-β-gal) staining (Fig. 8H). Additionally, R205K-expressing tumors displayed an elevation of p16^INK4a^ expression compared to that in the control LLC cells (Fig. 8I). Immunoblotting analysis indicated that there was much greater expression of senescence-related proteins (p53, p21, and p16^INK4a^) in R205K-expressing tumors than those in control LLC cells (Fig. 8O), further substantiating the impact of R205K on senescence. There was no significant difference in HIF1α expression between control LLC and R205K-expressing tumors (Fig. 8P). Likewise, no overt change was seen in HIF1α expression between cultured LLC and R205K-expressing LLC cells; HIF1α could be induced by DMOG to comparable levels in both types of cells (Fig. 8Q). Thus, it is unlikely that R205K exerts its role through HIF-1α.

VEGF expression (Fig. 8J) and micro-vessel density (Fig. 8K) within the R205K-expressing tumors were decreased compared with those in control LLC tumors. These data implied that inactivated EGLN3 in cancer cells blunted tumor angiogenesis. Compared to the control LLC tumors, there was much less infiltration of macrophages (Fig. 8L) and Treg cells (Fig. 8M) in R205K-expressing LLC tumors. There was no significant difference in the abundance of CD8^+^ T lymphocytes between the two groups (Fig. 8N). Taken together, inactivated EGLN3 hydroxylase in LLC cancer cells retarded tumor growth through programming an anti-tumor TME and enhancing senescence of LLC cells.

## Discussion

EGLN3 reportedly catalyzes the hydroxylation of several cancer-associated molecules, as exemplified by Erk3, p53, and HIF1α [7, 20, 27]. Our current study showed that inactivation of EGLN3 in host cells had minimal, if any, impact on the expression of HIF1α and p53. During the course of our study, a report demonstrated that EGLN3 stabilizes p53 via a hydroxylase-independent mechanism [29], which supports our finding. Our current work is also in line with the previous observation that EGLN3 hydroxylase exhibits a minimal effect on the hydroxylation and degradation of HIF1α in comparison to EGLN1 hydroxylase [28], although EGLN3 displays potential to catalyze the hydroxylation of HIF1α in some context [7]. Strikingly, our data highlight that EGLN3 hydroxylates and stabilizes Erk3, an attractive molecule orchestrating the initiation and progression and emerging as a therapy target of lung carcinoma.

One of the important findings of this work is our identification of new mechanism behind the regulation of Erk3 protein stability. Previous work revealed the implication of the Ub-proteasome pathway in Erk3 protein turnover [24]. To the best of our knowledge, we first demonstrated the critical role for the CMA in Erk3 degradation, revealing a novel mechanism regulating Erk3 protein stability. Our data clearly show that Erk3 is a substrate for the CMA. It is documented that the KFERQ-like motif is a characteristic of a CMA substrate protein [32]. Future studies are warranted to identify the KFERQ-like motif in the Erk3 protein. Importantly, our study uncovers the mechanism whereby EGLN3 stabilizes Erk3. Specifically, EGLN3 stabilizes Erk3 primarily through catalyzing Erk3 hydroxylation. Hydroxylation induced by EGLN3 protects against Erk3 interaction with LAMP2A and thereby its destruction via lysosomes.

This study is the first to describe the function and underlying mechanism for host EGLN3 hydroxylase activity in tumor growth. The TME plays critical roles in tumor initiation, progression, immune evasion, and resistance to therapy [3]. There are increasing interests in the study on the mechanism and regulation of macrophage recruitment, migration, and entrapment in the TME [6, 42]. Reduction of the content of macrophages, the most abundant immune cells in the TME, leads to the suppression of cancer growth [4]. We demonstrated that genetic or pharmacologic inactivation of EGLN3 significantly reduced macrophage content in the TME. EGLN3 inactivation impeded migration of macrophages both in vitro and in vivo. Inactivation of EGLN3 dramatically decreased the expression of NRP1, a key regulator of macrophage migration [6]. It is worth noting that Erk3 promotes NRP1 expression and macrophage migration. Future investigation will be warranted to determine whether EGLN3 hydroxylase regulates macrophage migration through the Erk3-NRP1 axis.

TAMs have been involved significantly in the immunosuppressive environment and angiogenesis [4]. TAMs contribute to creation of an immunosuppressive microenvironment primarily by blunting the recruitment of CD8^+^ T cells or by attracting Tregs to the tumor [43, 44]. Indeed, CD8^+^ T cells were increased in the TME while Tregs decreased when EGLN3 was inactivated genetically or pharmacologically. TAMs potentiate angiogenesis through multiple mechanisms. Adoptive transfer of macrophages with inactive EGLN3 activity curbed tumor angiogenesis. Emerging evidence assigned Erk3 a pro-angiogenic role [45]. Future studies are merited to determine whether EGLN3 regulates angiogenesis through Erk3. Taken together, deficiency of EGLN3 activity in the stromal cells (particularly macrophages in the current study) restrains cancer growth by mounting a robust anti-tumor immune response and restricting angiogenesis.

Previous studies have demonstrated that EGLN3 exhibits multifaceted functions [13–16, 46, 47]. We sought to explore the significance for induction of EGLN3 expression by hypoxia under which EGLN3 hydroxylase is substantially inactivated [8]. We showed that LLC cells with inactive EGLN3 exhibited an inhibition in growth in mice. Intriguingly, LLC cells expressing inactive EGLN3 acquired the characteristic hallmarks of cellular senescence. Senescence is a cell-intrinsic mechanism that is more important than forms of cell death for tumor suppression [48]. In this regard, our study revealed a novel cell-intrinsic mechanism for EGLN3 inactivation in regulating cancer growth. In addition, inactivation of EGLN3 hydroxylase ameliorates immunosuppressive environment. Taken together, our current work provides novel insights into intrinsic and extrinsic mechanisms coupling EGLN3 hydroxylase of cancer cells per se with the TME and cellular senescence.

In summary, this study sheds light on the role for the CMA-lysosome pathway in regulating Erk3 protein stability and provides novel insights into the mechanism underlying the stabilization of Erk3 by EGLN3. We first demonstrate that inactivation of EGLN3 hydroxylase in malignant and stromal cells protects against cancer by orchestrating the reciprocal interplays between cancer cells and the TME. These findings may offer impetus to develop more potent and selective inhibitors for EGLN3 hydroxylase in order to pave new avenue to effective cancer treatment.

## Materials And methods

### DNA constructs

To make vectors expressing full-length or different fragments of Erk3 with a C –terminal Flag tag, PCR amplification was performed using *pfu* polymerase and the amplicons were inserted into the BamH1 site of in p3xflag-CMV-13 (Sigma). Constructs expressing full-length or different regions of Erk3 with a N-terminal GST tag were produced by subcloning the PCR products into pEBG. The lentivirus expressing Erk3 was engineered by cloning Erk3 cDNA into pLvx-t2a-mecherry. The plasmid expressing LAMP2A was construct by subcloning the full-length LAMP2A cDNA into p3xflag-CMV-13. Flag-tagged Erk3 was a gift from Dr. A. von Kriegsheim [20]. The expression vector for TFEB was provided by Dr. W. Liu [49]. The HSC70 expression plasmid was a gift from Dr. Q. Lei [50]. Other plasmids used in this study were described in our previous publications [14, 15, 25, 26, 51]. All the constructs were verified by DNA sequencing.

### Antibodies

anti-FLAG M2 (Sigma, F1804), anti-HA.11 (BioLegend, 901501), anti-Myc (clone 9E10, Santa Cruz Biotechnology, sc-40), anti-GST (Yeasen, 30902ES60), anti-NRP1 (Cell Signaling Technology, #3725), anti-NRP2 (Cell Signaling Technology, #3366), anti-EGLN3 (Novus, NB100–303), anti-Erk3 (Santa Cruz Biotechnology, sc-365234), anti-arginase1 (Cell Signaling Technology, #9819), anti-α-tubulin (Sigma, T6074), anti-β-actin (Yeasen, 30101ES60), anti-GAPDH (Immunoway, YM3029), anti-CD31 (Abcam, ab28364), anti-Mac3 (BD Phmarmingen, 550292), anti-CD8 (InVitrogen, #14–0081–82), anti-F4/80 (Abcam, ab6640), anti-CD25 (Servicebrio, GB11612), anti-VEGF (Novus Biologicals, NB100–664), anti-PCNA (Servicebrio, GB11010), anti-cleaved caspase-3 (Cell Signaling Technology, #9661), anti-ubiquitin (Santa Cruz Biotechnology, sc-8017), anti-MMP2 (Abcam, ab37150), anti-phospho-IкBα (Cell Signaling Technology, #2859), anti-iNOS (Cell Signaling Technology, #2982), anti-p53 (Santa Cruz Biotechnology, sc-126), anti-LAMP2 (Santa Cruz Biotechnology, sc-18822), anti-p16 (Santa Cruz Biotechnology, sc-1661), anti-p21 (Santa Cruz Biotechnology, sc-817), normal mouse IgG1 (Santa Cruz Biotechnology, sc-3877), and anti-mouse IgG(H + L) (Jackson ImmunoResearch, 715-585-151).

### Site-directed mutagenesis

Site-directed mutagenesis of Erk3 was conducted to generate an P25A substitution mutation using Quick-Change^TM^ Site-directed Mutagenesis kit (Agilent Technologies) with the mutagenic primers as described previously [20, 51]: 5’-CTAGGTATATGGACTTAAAAGCCTTGGGTTGTGGAGGCAATG-3’ and 5’-CATTGCCTCCACAACCCAAGGCTTTTAAGTCCATATACCTAG-3’. The mutation was verified by DNA sequencing.

### Cell culture and transfection

Human embryonic kidney 293 T (HEK293T) cells, A549, Hep3B, LLC, and Jurkat T leukemia cells were obtained from ATCC (Manassas, VA). HEK293T, Hep3B, LLC, and A549 cells were maintained in Dulbecco’s Modified Eagle’s Medium supplemented with 10% heat-inactivated fetal bovine serum (FBS). Jurkat T cells were maintained in RPMI 1640 medium supplemented with 10% FBS. All cell lines were verified to be mycoplasma-free. Cells were transfected using Lipofectamine 2000 reagent (Invitrogen) following the manufacturer’s instructions.

### Preparation of bone marrow-derived macrophages (BMDMs)

Mouse BMDMs were prepared by flushing the marrow from the tibiae and femorae obtained from 6–8-week-old mice as described previously [52]. Cells were then differentiated into macrophages by culturing in DMEM media supplemented with 10% FBS and 1% penicillin/streptomycin in the presence of 20% L929-conditioned media for 7 days.

### RNA interference

Freshly isolated mouse bone marrow derived macrophages were transfected by Lipofectamine 3000 reagent (Invitrogen) with 100 nM of Erk3 or control small interfering RNA (siRNA), both of which were purchased from Santa Cruz Technology. After transfection, cells were cultured for 48 h in DMEM containing 10% FBS before further experiments. Smart-pool EGLN3 and control siRNAs were purchased from Dharmacon. LAMP2A siRNAs (5’-CGCUAUGAAACUACAAAUA-3’ and 5’-GCUCUACUUAGACUCAAUA-3’) and control siRNAs were purchased from RIBOBIO (Guangzhou, China). EGLN3 and LAMP2A siRNAs (50–75 nM) were transfected into cells using Lipofectamine 2000 reagent (Invitrogen) as described previously [25]. siRNA transfection efficiency was verified by immunoblotting.

### Lentiviral infection

For generation of lentivirus particles, HEK293T cells were co-transfected using Lipofectamine 2000 (Invitrogen) with lentiviral vector and packaging plasmids as follows: plvx-Erk3 (3 μg), plp1 (1 μg), plp2 (1 μg) and vsvg (1 μg). After 24 h and 48 h posttransfection, the supernatants containing lentiviral particles were harvested and filtered through 0.45-μm filters. After infection of lentivirus for 48 h, macrophages were harvested.

### Reverse transcription-PCR

Total RNA was extracted from the cells using Trizol reagent (Invitrogen). Reverse transcription of mRNA was performed using a RevertAid First Strand cDNA Synthesis Kit (Thermo Fisher Scientific). For analysis of the expression of EGLN3 or EGLN3R205K, conventional reverse transcription-PCR (RT-PCR) was performed using EGLN3 specific primers. For quantitative PCR (qPCR) analysis of gene expression, amplification was conducted using a FastStart Universal SYBR Green Master (Roche) and run on a Real‐time PCR System (ABI‐7000). The Ct values for target genes and the reference gene, GAPDH, were recorded. Fold induction was calculated using the ΔΔCt method. Primer sequences are available upon request.

### Immunoblotting

Immunoblotting was carried out as previously described [14, 15, 25, 26, 51]. Cells were harvested in Triton X-100-based lysis buffer (20 mM Tris [pH 7.4], 120 mM NaCl, 5 mM EDTA, 1% Triton X-100, 10% glycerol, 1 mM phenylmethylsulfonyl fluoride (Sigma) and complete protease inhibitor mixture (Roche Applied Science) for 0.5 h at 4 °C. The debris was removed by centrifugation at 15,000 *g* for 30 min at 4 °C. The soluble fractions were recovered and proteins were quantified using the Micro BCA^TM^ protein assay reagent kit (Pierce). Cellular proteins were resolved on SDS-PAGE and electroblotted onto a polyvinylidene difluoride (PVDF) membrane (Bio-Rad) or a nitrocellulose membrane (Bio-Rad). Following blocking, the membrane was incubated with an appropriate primary antibody and then incubated with a corresponding sheep anti-mouse IgG or donkey anti-rabbit IgG conjugated to horse radishperoxidase. The blots were developed by ECL or ECL Plus method.

### Immunoprecipitation

Immunoprecipitation was conducted as previously described [51]. Cells were solubilized in lysis buffer [25]. The precleared lysates were incubated with the corresponding antibody (about 1–1.5 μg each) in the presence of 20 μl of Protein A/G Agarose (Pierce) overnight with constant agitation. After extensive washing, the immunoprecipitates were subjected to immunoblotting.

### GST and His pull-down assays

GST and His pull-down assays were performed as previously described [51]. HEK293T cells were transfected as indicated in the figure legends. At 24 h posttransfection, cells were collected in NETN buffer (20 mM Tris–HCl [pH 8], 100 mM NaCl, 1 mM EDTA, 0.5% IGEPAL CA-630, and protease inhibitors. About 300–700 μg of total cell lysates were mixed with 15 μl of Glutathione-Sepharose 4B beads (for GST pull down assay; Yeasen, Cat#: 2050 7ES10) or nickel-nitrilotriacetic acid-agarose (for His pull down assay; YESEAN, Cat#: 30902ES20) in NETN buffer with protease inhibitors. Beads were subjected to 2–3 washes with NETN buffer containing protease inhibitors for 20 min and 2–4 washes with Buffer B (137 mM NaCl, 2.7 mM KCl, 8.1 mM Na_2_HPO4, 14.7 mM KH_2_PO_4_, pH 7.4) containing protease inhibitors at 4 °C for 20 min. The complexes were then eluted with SDS sample buffer and detected by immunoblotting.

### Immunostaining

Immunostaining was conducted as previously described [15, 51]. A549 cells were co-transfected with the constructs expressing Flag-p53 and EGLN3 or EGLN3R205K. A total of 24 h post-transfection, cells were fixed in 4% paraformaldehyde for 10 min at room temperature (RT), permeabilized in PBST (PBS containing 0.2% Triton X-100) for 5–10 min at RT, and blocked in PBS with 1% bovine serum albumin. Cells were incubated with an anti-Flag monoclonal antibody at 4 °C overnight, followed by incubation with Texas Red-conjugated anti-mouse IgG (Santa Cruz Biotechnology) for 45 min at RT. Following extensive washing with PBST, cells were probed with an rabbit anti-EGLN3 (Novus) overnight at 4 °C, followed by incubation with fluorescein isothiocyanate conjugated anti-rabbit IgG (Molecular Probes) for 45 min at RT. Cells were visualized by a fluorescent microscope.

### Cycloheximide chase experiment

Cycloheximide chase experiment was conducted as previously described [51]. In brief, 293 T cells grown onto 35-mm plates were transfected with the constructs expressing wild-type (WT) EGLN3, hydroxylase-inactive mutant EGLN3 (R205 K) or empty vector. After 24 h, cells were treated with 30 μg/mL of protein synthesis inhibitor cycloheximide (CHX, Sigma) for the indicated time points, when the cells were harvested. Cellular extracts were normalized for total protein content and subjected to immunoblotting using the indicated antibodies.

### Generating apoptotic Jurkat cells

Jurkat cells were seeded at a density of 1 × 10^6^/mL in fresh cell culture medium and labeled with Calcein AM at 37 °C for 2 h. Labeling was monitored under a fluorescence microscope. After thoroughly washing to remove unincorporated dye, cells were resuspended into fresh culture media. For induction of apoptosis, staurosporine (STA) was added to the labeled cells to a final concentration of 1 μM for 2 h.

### In vitro efferocytosis assay

About 2.5 × 10^5^ BMDMs were seeded onto 24-well tissue culture plate and grown overnight. Around 5 × 10^5^ apoptotic Jurkat T cells were loaded onto macrophages and incubated at 37 °C for 45 min. Level of engulfment was monitored under fluorescent scope. Apoptotic cells were washed away with PBS. The cells were fixed with 4% paraformaldehyde (PFA) for 10 min. The images were acquired and efferocytosis quantitated.

### Macrophage polarization assay

Bone marrow derived macrophages were treated with lipopolysaccride (LPS, 100 ng/mL) and interferon γ (IFNγ, 20 ng/mL) to induce macrophages polarization towards an M1 phenotype or with interleukin 4 (IL-4, 20 ng/mL) to induce M2 polarization. About 48 h later, cells were harvested for qRT–PCR or immunoblotting.

### Cell proliferation assay

Cell viability was determined by CCK8 assay. Briefly, cells were seeded in 96-well plates (5 × 10^3^ cells/well) and treated as indicated in the Figure Legends. CCK8 was added into the wells for 3 h at indicated times. The absorbance in each well at wavelength of 450 nm (OD450) was measured with a Thermomax microplate reader.

### In vitro cell migration assay

In vitro migration of macrophages was assessed using a wound-healing assay as described previously [53] with minor modifications. In brief, 1 × 10^6^ macrophages were seeded in 35-mm culture dishes and incubated in the complete medium overnight. The confluent monolayer of cells was scratched straightly with a 100-μl pipette tip, and the cellular debris rinsed away with PBS. Migration was visualized under a microscope and the images captured at the indicated time points. The cells in the denuded regions were counted and statistically analyzed.

### In vivo migration assay of murine macrophages

To demonstrate the effect of EGLN3 activity on mouse macrophage migration in vivo, WT and KI mice were injected intraperitoneally with 1 ml of 2.5% thioglycollate. Mice were sacrificed 72 h later and the peritoneal cavity was lavaged with 5 ml of PBS. The percentage of macrophages in the lavage were quantified by flow cytometry with PE-conjugated anti–F4/80 antibody.

### Flow cytometry

To assay for the content of macrophages in the peritoneal lavage, cells were stained with F4/80, or negative control antibody. All samples were analyzed using a FACS Fortessa cytometer (BD Biosciences). Data were analyzed using FlowJo software (Treestar).

### Senescence-associated β-galactosidase (SA-β-Gal) staining

To visualize senescent cells, SA-β-Gal staining was performed according to the protocol of the manufacturer (Beyotime Biotechnology Ltd., Shanghai, China). For SA-β-Gal staining of tumor tissues, the frozen sections of tumor tissues were incubated in SA-β-gal staining solution at 37 °C for 20 h. For quantification of senescent cells, SA-β-Gal^+^ blue cells in six randomly selected fields at ×200 magnification were counted. The senescent cells were expressed as the percentage of SA-β-Gal^+^ cells within randomly selected fields.

### Immunohistochemistry (IHC)

Mouse tissues were fixed in 60% methanol and 10% acetic acid in H_2_O (vol/vol) and embedded in paraffin. Tissue sections (5 μm) were subjected to deparaffinization and rehydration, followed by treatment with 3% hydrogen peroxide solution for 20 min to block endogenous peroxidase activity. Antigen retrieval was carried out by treatment of the slides with EDTA (pH 8.0) or 10 mM sodium citrate buffer (pH 6.0) by microwaving for 10 min. The samples were blocked with 5% fetal bovine serum in 0.1% PBS/bovine serum albumin and incubated with the indicated primary antibody overnight at 4 °C. The standard streptavidin–biotin linked horseradish peroxidase technique was then conducted with 3,30-diaminobenzidine tetrahydrochloride being used for the development of peroxidase activity. The sections were counterstained with haematoxylin.

For quantification of immunostaining results, we counted the marker-positive cells or vessel numbers based on CD31 staining of at least ten random 40X fields per mouse and 4–5 mice per group were analyzed, which were conducted manually or using the Cell Counter function in NIH ImageJ. We then averaged the results over the number of counted fields. Data in graphs were presented as mean ± SEM. To minimize possible effects of a reaction to necrotic cell debris, cells immediately adjacent to necrotic areas were not included in the counts [54], if possible. All counts were performed in a case-blinded manner by two well-trained individuals. The analyses between the two investigators showed high concordance and the numbers were averaged to yield the reported results.

### Generation of EGLN3R205K knock-in mice

EGLN3R205K knock-in (KI) mice were generated at the Genomic Center of the University of Rochester animal facility. Mice harboring EGLN3R205K mutation were generated by CRISPR/Cas9-mediated genome editing. Briefly, the fragment for EGLN3 gRNA (ATGCCACCAGGTAAGAGCTG) was inserted into the gRNA cloning vector. Both the EGLN3 gRNA and Cas9 mRNA were generated by in vitro transcription. The oligo donor with targeting sequence, flanked by 120 bp homologous sequences combined on both sides (5’-TTCTGTTCTTCTGGTCAGACCGCAGGAATCCACATGAAGTCCAGCCCTCCTATGCCACGAAGTAAGAGCTGGGGCCACAGTTCCTCTTCCAGGGTGCATACAAACCCCAGATCCCCG-3’) was synthesized (Integrated DNA Technologies). To obtain EGLN3R205K mice, the EGLN3 gRNA, Cas9 mRNA and oligo donor were co-injected into fertilized eggs. The pups were genotyped by PCR using primers (R205K Forward primer: 5’-GCCCTCCTATGCCACGAA-3’; WT Forward primer: 5’-CCTCCTATGCCACCAGGTAA-3’; Reverse primer: 5’-CAGTATGCACAACTCACAGGA-3’). The male pups harboring EGLN3 mutation were mated with C57BL/6 female mice. Heterozygotic mice for the mutated EGLN3 locus were intercrossed to generate homozygotic EGLN3R205K mice. EGLN3R205 K KI mice on a C57BL/6 background were maintained in specific pathogen free (SPF) level, independent ventilation cage (IVC) environment on a regular light-dark cycle (12 h light, 12 h dark).

### Tumor xenografts and tumor volume measurement

All mouse procedures and experiments for this study were approved by the Institutional Animal Care and Use Committee of Renmin Hospital at the Hubei University of Medicine. C57Bl/6 mice were purchased from the Jackson Laboratory. Mice were randomly divided into two groups for each animal experiment. All mice were sex- and age-matched. Equal amounts of male and female animals were used in each study. A total of 80 mice were used in our animal studies. We performed two separate experiments to analyze the characteristics of LLC tumor in WT and KI mice. The investigators were blinded to the group allocation during the experiment and/or when assessing the outcome. The animals that were injured, free of tumor, or died during the course of experiments were excluded from the analysis.

Xenograft transplantations were performed in a blind manner in 6–8-week-old WT (C57BL/6) and EGLN3R205K knock-in mice according to the institutional guidelines and permissions for animal experiments, obtained from the regional authorities of the Hubei University of Medicine. For subcutaneous tumor experiments, 5 × 10^5^ murine LLC lung carcinoma cells in 0.1 ml PBS were injected subcutaneously into the flanks of 6–8-week-old WT and R205 K knock-in mice. Tumor dimensions were measured once when tumors were palpable. The tumor size was measured with a caliper and the tumor volume was calculated using the formula: tumor volume = 0.5 × a^2^ × b (where a is the short tumor diameter and b is the long tumor diameter).

Co-injection of macrophages and LLC cells were conducted as described previously [55]. Macrophages (2.5 × 10^5^) will be mixed with 6 × 10^5^ LLC cells in 100 μl PBS and were co-injected subcutaneously into wild type, 6–8-week-old C57BL/6 mice. Tumor size was measured from the fifth day after injection. Tumor volumes were measured and calculated.

At the end of the experiment, mice were anesthetized by isoflurane. The mice were perfused via the left ventricle with 0.9% saline supplemented with heparin (50 U/mL), followed by another perfusion with 4% paraformaldehyde solution. The tumors were harvested and embedded in paraffin or optimal cutting temperature compound (OCT, Tissue-Tek) and frozen in −80 °C for cryostats tissue sectioning. Tumors dissociated were subjected to immunohistochemical, FACS, and immunoblotting analyses.

### Statistical analysis

Data analyses were performed using GraphPad Prism 8.0 (GraphPad Software). Normal distribution was evaluated using the Shapiro-Wilk test. The variance was similar between the groups. Two-tailed Student’s *t* test was applied to assess statistical differences between the two groups. Data are expressed as the mean ± SEM (standard error of the mean). The values of *p < 0.05, **p < 0.01, ***p < 0.001, and ****p < 0.0001 were considered statistically significant.

## Supplementary information

Supplementary Figure Legends

Supplementary Fig. 1 EGLN3 stabilized the tumor suppressor p53 independently of its hydroxylase activity.

Supplementary Fig. 2 EGLN3 stabilized Erk3 by antagonizing lysosomal degradation in a hydroxylase-dependent fashion.

Supplementary Fig. 3 Erk3 is a novel substrate for the chaperon-mediated autophagy.

Supplementary Fig. 4 Characterization of Erk3 interaction with HSC70 and LAMP2A.

Supplementary Fig. 5 EGLN3, but not hydroxylase-inactive mutant R205K, antagonized Erk3 interaction with HSC70 and LAMP2A.

Supplementary Fig. 6 Hydroxylation enhanced the stability of the Erk3 protein.

Supplementary Fig. 7 EGLN3 selectively stabilized the Erk3 protein.

Supplementary Fig. 8 The effects of EGLN3 inactivation on the properties of macrophages.

Supplementary Fig. 9 The effects of DMOG on the properties of macrophages.

Supplementary Fig. 10 Expression of hydroxylase-inactive EGLN3 had no effects on proliferation and apoptosis of LLC lung cancer cells.
