# Supplementary material for: Inactivation of EGLN3 hydroxylase facilitates Erk3 degradation via autophagy and impedes lung cancer growth
Source: Oncogene. 2022 Feb 5;41(12):1752–66. doi: 10.1038/s41388-022-02203-2 (PMC8933280; doi:10.1038/s41388-022-02203-2)
Supplement: Supplementary file 1 — Supplementary Figure Legends [file 41388_2022_2203_MOESM1_ESM.docx]

**Inactivation of EGLN3 hydroxylase facilitates Erk3 degradation via autophagy and impedes lung cancer growth**

Ying Jin^1,4, *, #^, Yamu Pan^1, *^, Shuang Zheng^1, 3^, Yao Liu^1^, Jie Xu^1^, Yazhi Peng^1^, Zemei Zhang^2,1^, Yadong Wang^1^, Yulian Xiong^1^, Lei Xu^1^, Kaiyu Mu^1^, Suwen Chen^1^, Fei Zheng^1^, Ye Yuan^1^, Jian Fu^1,4, #^

**Supplementary Figure Legends**

**Supplementary Fig. 1 EGLN3 stabilized the tumor suppressor p53 independently of its hydroxylase activity.**

**A** IB analysis of the effect of EGLN3 knockdown on p53 expression in A549 lung cancer cells transfected with control (-) or EGLN3 (+) siRNA. **B** IB and RT-PCR analysis of the impact of EGLN3 or R205K on p53 expression in Hep3B liver cancer cells transfected with either EGLN3 or R205K. **C, D** His pulldown analysis of p53 interaction with EGLN3 or R205K in A549 cells transfected with the indicated plasmids. **E** co-localization of p53 and EGLN3 or R205K in A549 cells transfected with Flag-p53 and EGLN3 or R205K. Immunofluorescence was conducted with anti-EGLN3 and anti-Flag.All experiments were repeated three times.

**Supplementary Fig. 2 EGLN3 stabilized Erk3 by antagonizing lysosomal degradation in a hydroxylase-dependent fashion.**

**A** GST pulldown analysis of the interaction between Erk3 and EGLN3. Cell lysates prepared from HEK293T cells transfected with the indicated plasmids were incubated with Glutathione-Sepharose 4B beads and then immunoblotted with anti-Flag (n=3). **B** IB analysis of cell lysates prepared from HEK293T cells transfected with the indicated plasmids (n=3). **C** Cycloheximide chase experiment was conducted to analyze the effect of EGLN3 on Erk3 stability. HEK293T cells transfected with the indicated constructs were exposed to CHX for various time points, followed by IB analysis of cell lysates (n=3). **D** The *in vivo* ubiquitination assay was conducted to evaluate the effect of EGLN3 on Erk3 ubiquitination (n=4). **E** HEK293T cells were transfected with EGLN3 or R205K. IB analysis was performed to examine Erk3 expression in HEK293T cells exposed to Bafilomycin A1 (n=3). IB, immunoblotting; GST, glutathione S-transferase; PD, pulldown; CHX, cycloheximide; Ub, ubiquitin.

**Supplementary Fig. 3 Erk3 is a novel substrate for the chaperon-mediated autophagy.**

**A, B** Sub-confluent HEK293T (**A**) and Hep3B (**B**) cells were cultured in the presence (+) or absence (-) of 10% FBS for the indicated time points. IB was carried out to monitor Erk3 expression. **C** IB analysis of cell lysates extracted from HEK293T cells exposed to etoposide for the indicated time points. **D, E** IB analysis of cell lysates from HEK293T cells transfected with indicated constructs; **F** IB analysis of cell lysates extracted from Hep3B (left panel) and HEK293T (right panel) cells transfected with (+) or without (-) myc-TFEB. **G** IB analysis of lysates prepared from HEK293T cells or those transfected with control or LAMP2A siRNAs. Blots were scanned and densitometry was performed using ImageJ software. Shown is the standard curves. FBS, fetal bovine serum; PC, positive control; HSC70, heat shock cognate protein of 70 kDa; LAMP2A, lysosome-associated membrane protein type 2A; TFEB, transcription factor EB; IB, immunoblotting. All experiments were repeated three times.

**Supplementary Fig. 4 Characterization of Erk3 interaction with HSC70 and LAMP2A.**

**A-E** Co-immunoprecipitation analysis of the interaction between HSC70 and Erk3. Cell lysates prepared from HEK293T cells transfected with the indicated plasmids were immunoprecipitated with anti-Flag or mIg (as a control) and then immunoblotted with the indicated antibodies. **F-I** GST pulldown analysis of the interaction between HSC70 and Erk3. Cell lysates extracted from HEK293T cells transfected with the indicated plasmids were incubated with Glutathione-Sepharose 4B beads and then immunoblotted with the indicated antibodies. **J** Co-immunoprecipitation analysis of the interaction between LAMP2A and Erk3. Cell lysates prepared from HEK293T cells transfected with the indicated constructs were immunoprecipitated with anti-Flag and then immunoblotted with anti-myc. **K, L** GST pulldown analysis of the interaction between LAMP2A and Erk3; **M, N** GST pulldown assay for the HSC70 and LAMP2A interaction with full-length Erk3, Erk3 (1-340) or Erk3 (341-721). HSC70, heat shock cognate protein of 70 kDa; LAMP2A, lysosome-associated membrane protein type 2A; IP, immunoprecipitation; IB, immunoblotting; PD, pulldown; IgH, the heavy chain of IgG; mIg, mouse IgG; GST, glutathione S-transferase. All experiments were repeated three times.

**Supplementary Fig. 5 EGLN3, but not hydroxylase-inactive mutant R205K, antagonized Erk3 interaction with HSC70 and LAMP2A.**

**A, B** GST pulldown analysis of the effect of EGLN3 on Erk3 interaction with HSC70 and LAMP2A. **C, D** GST pulldown analysis of the effect of EGLN3 on Erk3(1-340) interaction with HSC70 and LAMP2A. **E, F** GST pulldown analysis of the effect of R205K on Erk3 interaction with HSC70 and LAMP2A. **G** GST pulldown analysis of Erk3 or hydroxylation-resistant mutant Erk3(P25A) interaction with Flag-EGLN3 or Flag-R205K. HSC70, heat shock cognate protein of 70 kDa; LAMP2A, lysosome-associated membrane protein type 2A; IB, immunoblotting; PD, pulldown; GST, glutathione S-transferase. All experiments were repeated three times.

**Supplementary Fig. 6 Hydroxylation enhanced the stability of the Erk3 protein.**

HEK293T cells were transfected with Flag-Erk3 or Flag-Erk3P25A. Cells were exposed to cycloheximide for indicated times. IB was carried out to analyze the expression of Flag-Erk3 and Flag-Erk3P25A. Tubulin was used as the loading control (n=3). CHX, cycloheximide; IB, immunoblotting.

**Supplementary Fig. 7 EGLN3 selectively stabilized the Erk3 protein.**

**A** Co-immunoprecipitation analysis was performed to examine the interaction between Erk3 and EGLNs. Cell lysates prepared from HEK293T cells transfected with the indicated plasmids were immunoprecipitated with anti-Flag, followed by IB with anti-myc and -Flag. Cell lysates were monitored as indicated. **B** GST pulldown analysis was conducted to probe the interaction between Erk3 and EGLNs. Cell lysates from HEK293T cells transfected with the indicated plasmids were incubated with Glutathione-Sepharose 4B beads and then immunoblotted with the indicated antibodies. IP, immunoprecipitation; IB, immunoblotting; PD, pulldown; IgH, the heavy chain of IgG; GST, glutathione S-transferase. All experiments were repeated three times.

**Supplementary Fig. 8 The effects of EGLN3 inactivation on the properties of macrophages.**

**A** IB analysis of MMP2 expression in macrophages prepared from WT and KI mouse. **B** IB analysis was conducted to assess the influence of EGLN3 inactivation on staurosporine-induced expression of cleaved caspase-3 in macrophages prepared from WT and KI mouse. **C** CCK8 assay was performed to estimate the effect of EGLN3 inactivation on the proliferation of macrophages prepared from WT and KI mouse. **D** IB was carried out to explore the impact of EGLN3 inactivation on LPS-triggered IкBα phosphorylation in macrophages from WT and KI mouse. LPS, lipopolysaccride; STA, staurosporine; MMP2, matrix metalloproteinase 2; Casp3, Caspase 3; IB, immunoblotting. All experiments were repeated three times.

**Supplementary Fig. 9 The effects of DMOG on the properties of macrophages.**

**A** Wound assay was conducted to determine the effect of DMOG on the migration of macrophages. Shown are representative photographs at different time points after scratching. **B** IB analysis of PARP and cleaved caspase 3 expression in macrophages treated with STA or DMOG. **C** IB analysis of PCNA expression in macrophages treated with DMOG (+) or PBS (-). **D** CCK8 assay was conducted to evaluate the impact of DMOG on the proliferation of macrophages. DMOG, dimethyl oxalyglycine; STA, staurosporine; Casp3, Caspase 3; IB, immunoblotting. All experiments were repeated three times.

**Supplementary Fig. 10 Expression of hydroxylase-inactive EGLN3 had no effects on proliferation and apoptosis of LLC lung cancer cells.**

**A** IB analyzed the effect of EGLN3 or R205K on the expression of cleaved caspase 3 in LLC cells in response to STA. **B** Apoptosis occurred in LLC cells harboring R205K or the control LLC cells exposed to STA. **C** Comparison of the proliferation of LLC cells harboring R205K or the control LLC cells. STA, staurosporine; IB, immunoblotting; Casp3, Caspase 3. All experiments were repeated three times.
