# Supplementary figures and images for: Inactivation of EGLN3 hydroxylase facilitates Erk3 degradation via autophagy and impedes lung cancer growth
Source: Oncogene. 2022 Feb 5;41(12):1752–66. doi: 10.1038/s41388-022-02203-2 (PMC8933280; doi:10.1038/s41388-022-02203-2)

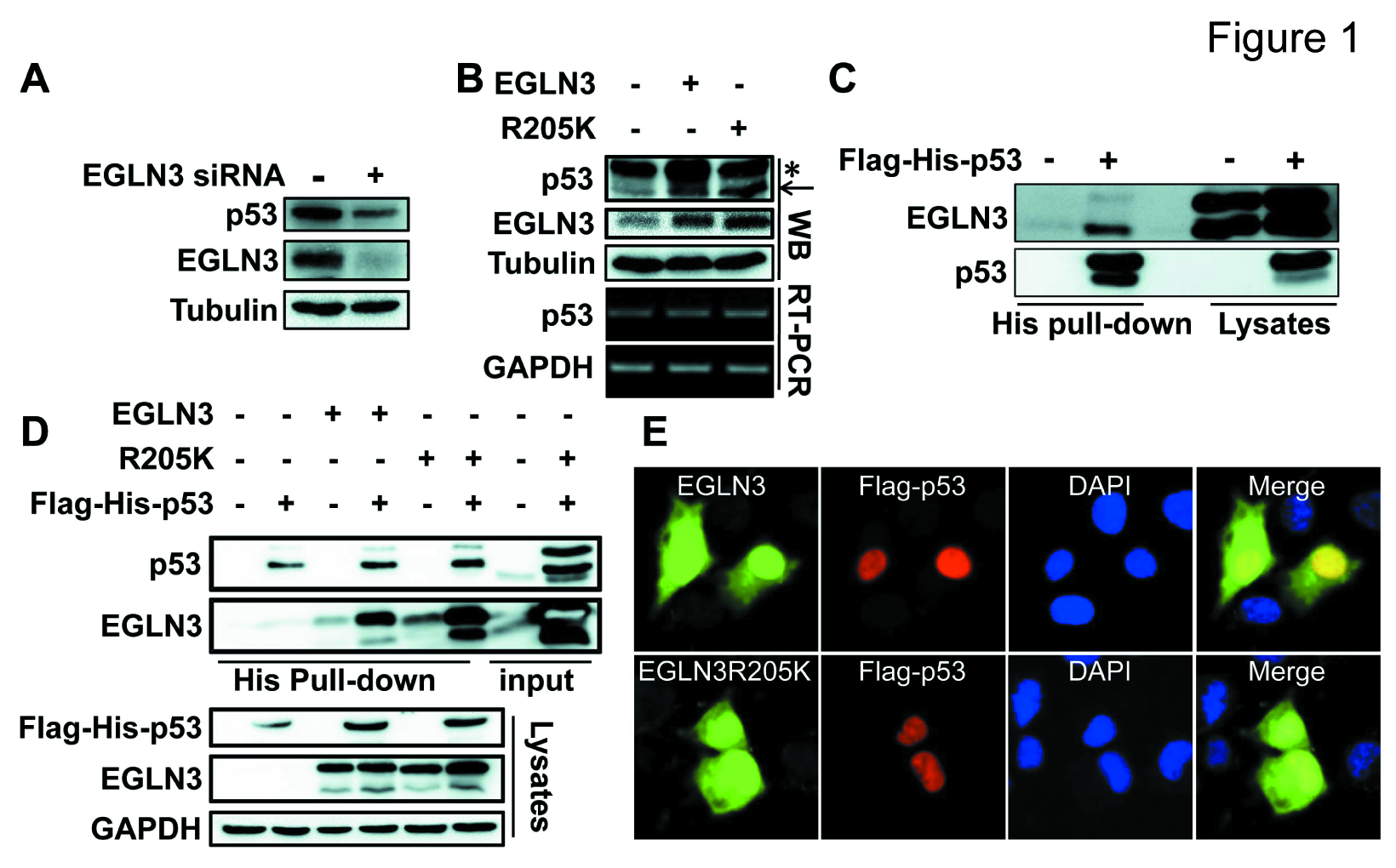

Supplement: Supplementary file 2 — Supplementary Fig. 1 EGLN3 stabilized the tumor suppressor p53 independently of its hydroxylase activity. [file 41388_2022_2203_MOESM2_ESM.tif]

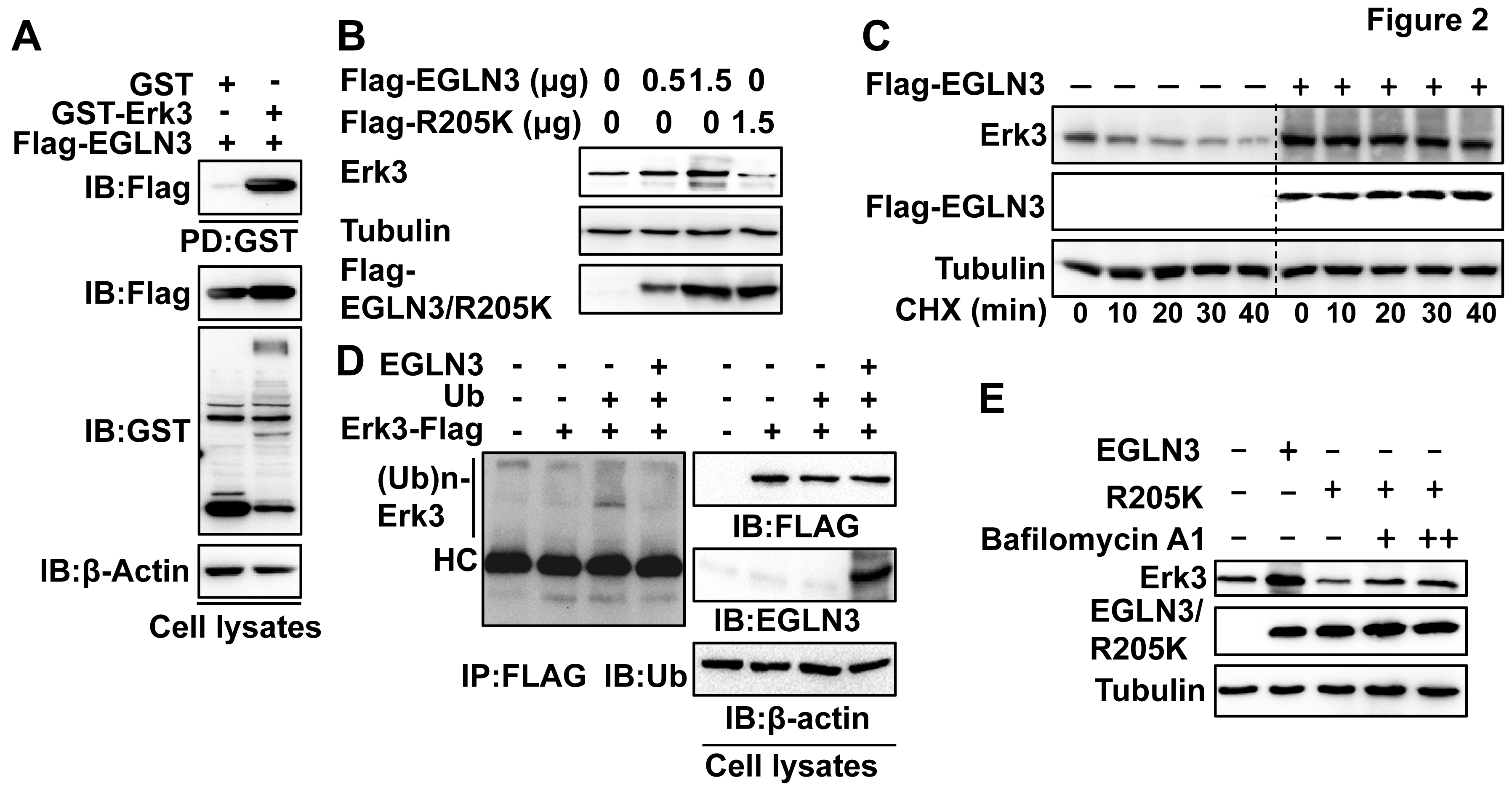

Supplement: Supplementary file 3 — Supplementary Fig. 2 EGLN3 stabilized Erk3 by antagonizing lysosomal degradation in a hydroxylase-dependent fashion. [file 41388_2022_2203_MOESM3_ESM.tif]

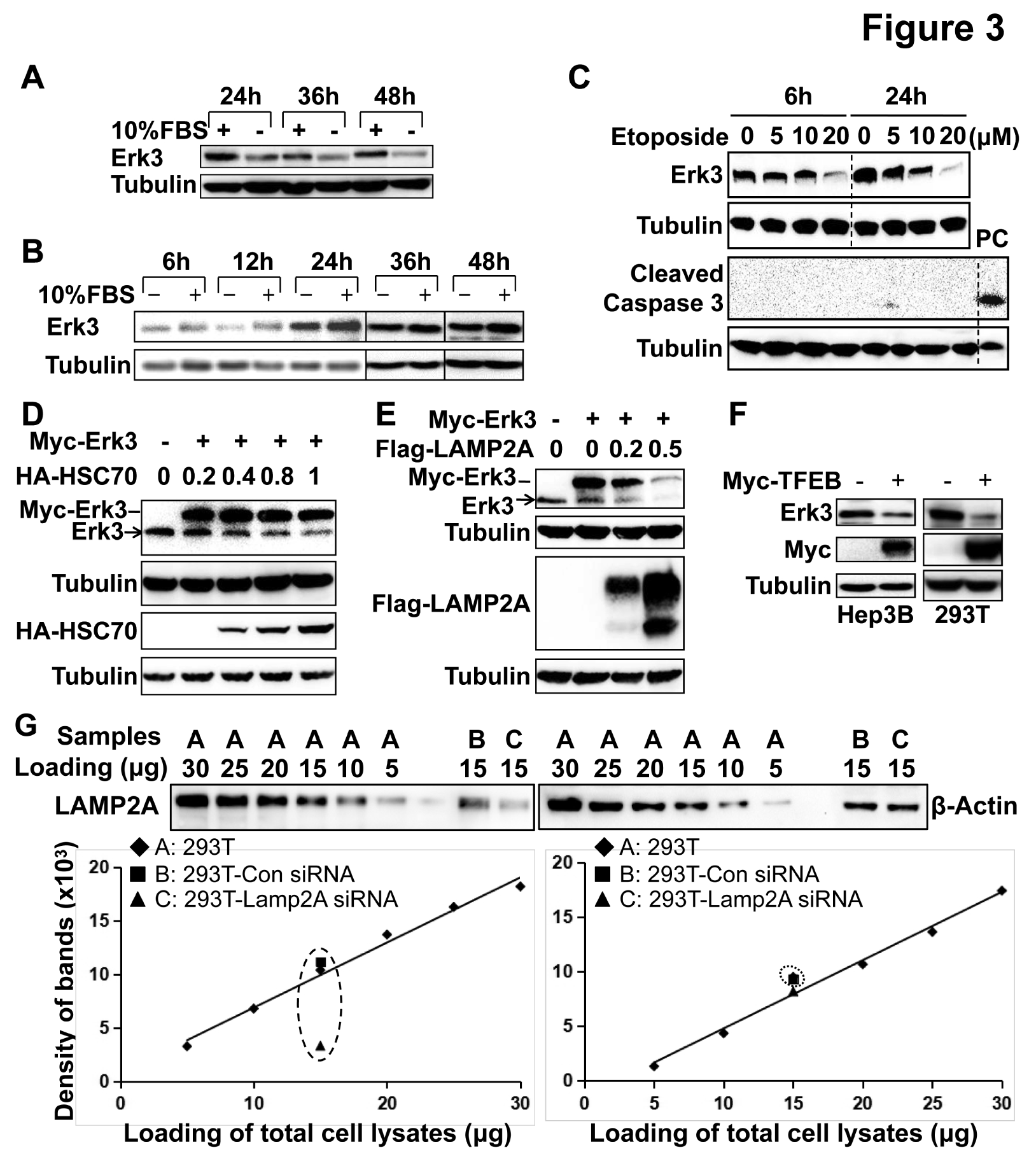

Supplement: Supplementary file 4 — Supplementary Fig. 3 Erk3 is a novel substrate for the chaperon-mediated autophagy. [file 41388_2022_2203_MOESM4_ESM.tif]

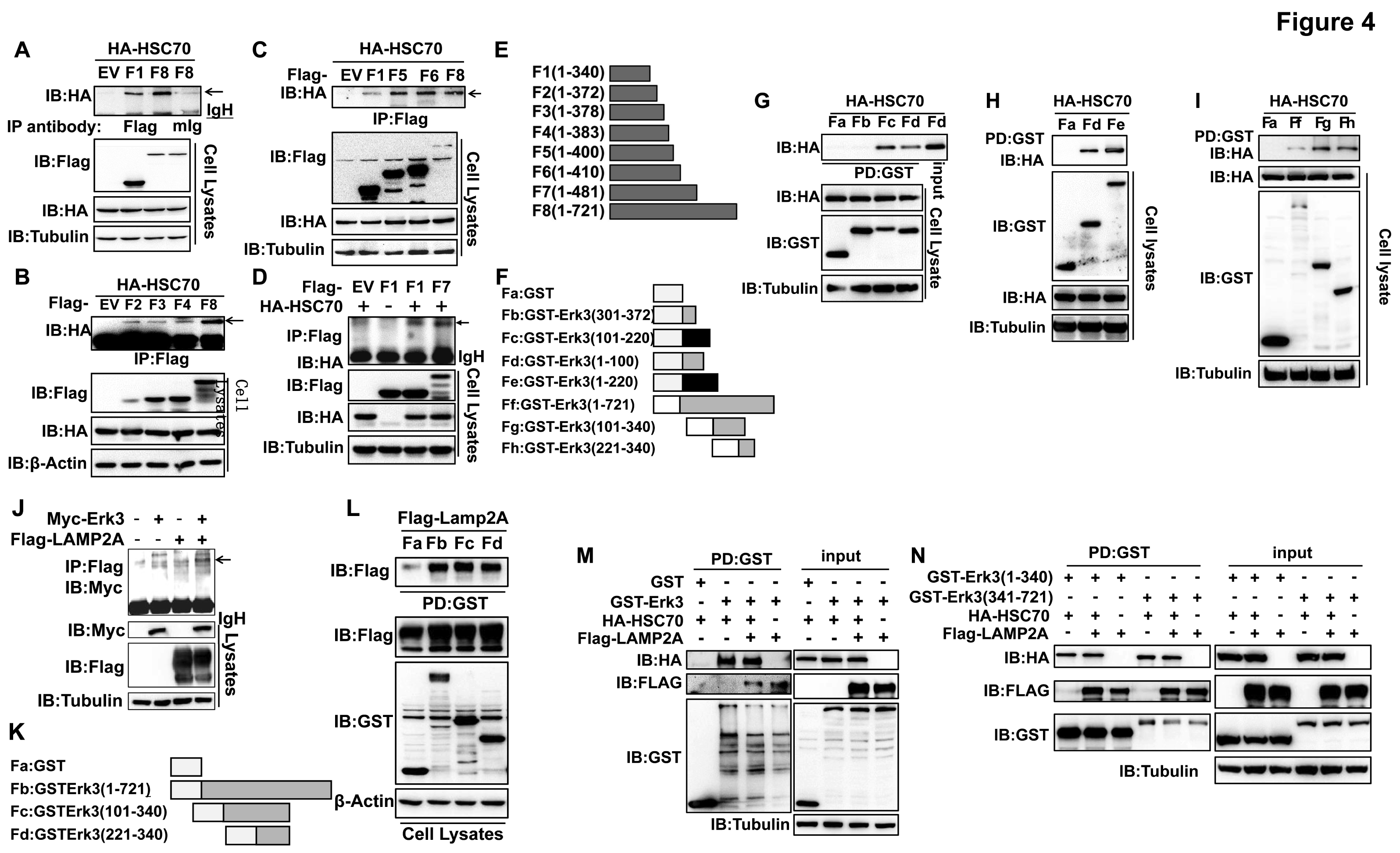

Supplement: Supplementary file 5 — Supplementary Fig. 4 Characterization of Erk3 interaction with HSC70 and LAMP2A. [file 41388_2022_2203_MOESM5_ESM.tif]

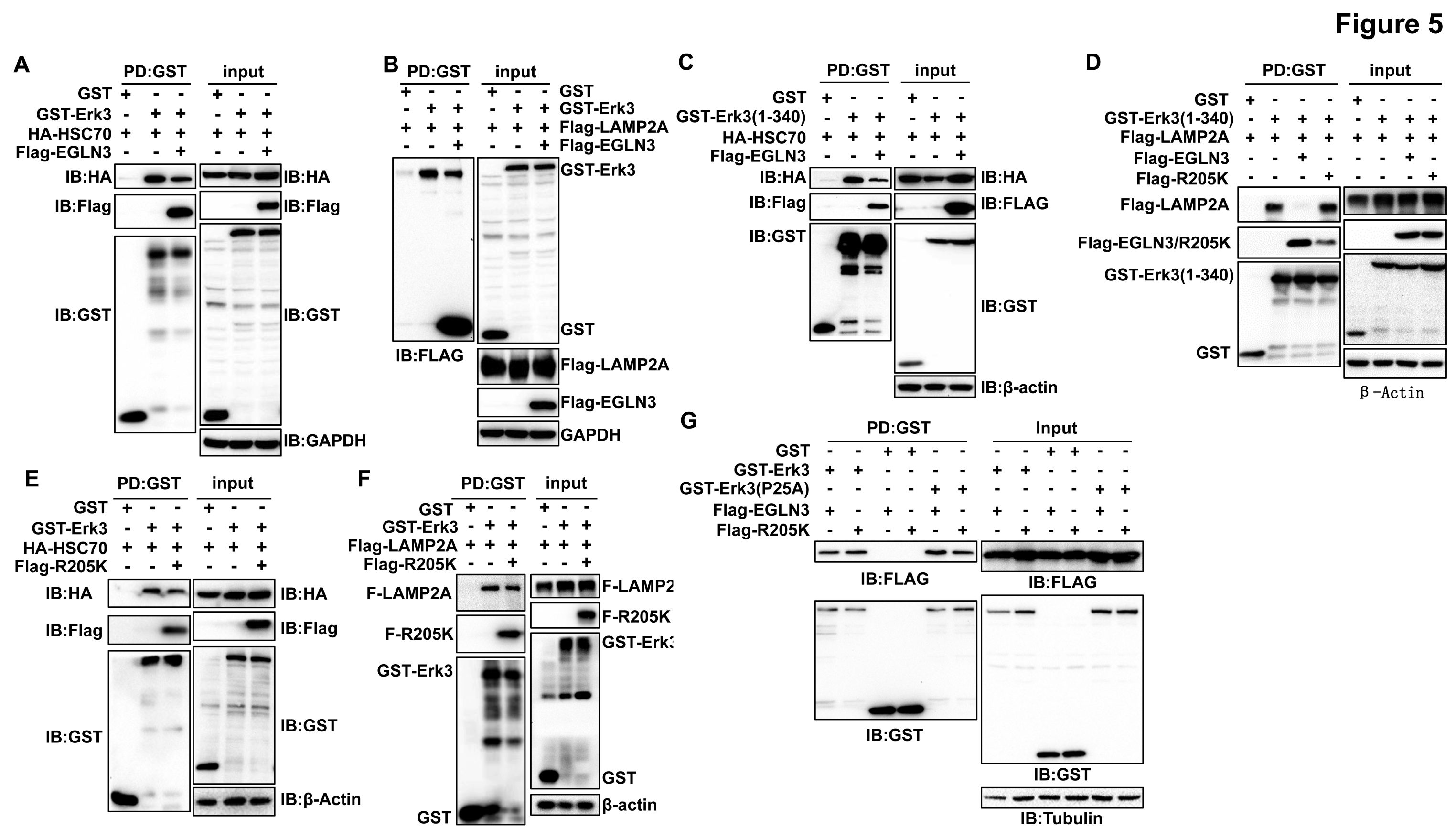

Supplement: Supplementary file 6 — Supplementary Fig. 5 EGLN3, but not hydroxylase-inactive mutant R205K, antagonized Erk3 interaction with HSC70 and LAMP2A. [file 41388_2022_2203_MOESM6_ESM.tif]

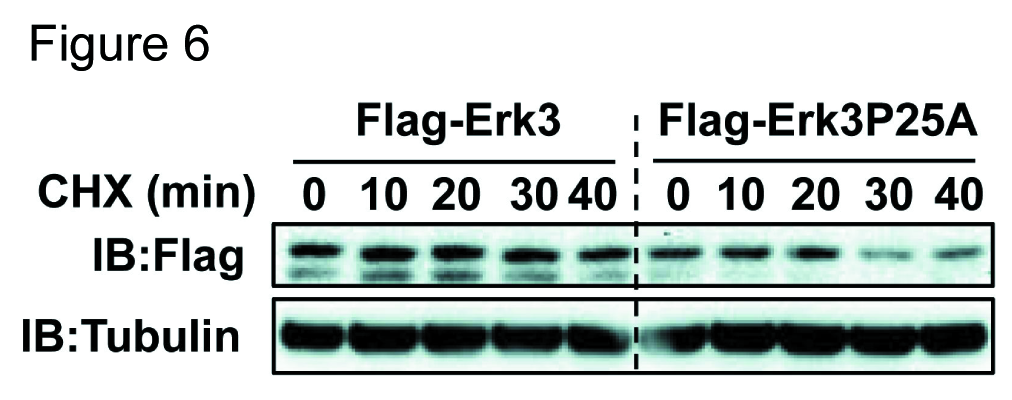

Supplement: Supplementary file 7 — Supplementary Fig. 6 Hydroxylation enhanced the stability of the Erk3 protein. [file 41388_2022_2203_MOESM7_ESM.tif]

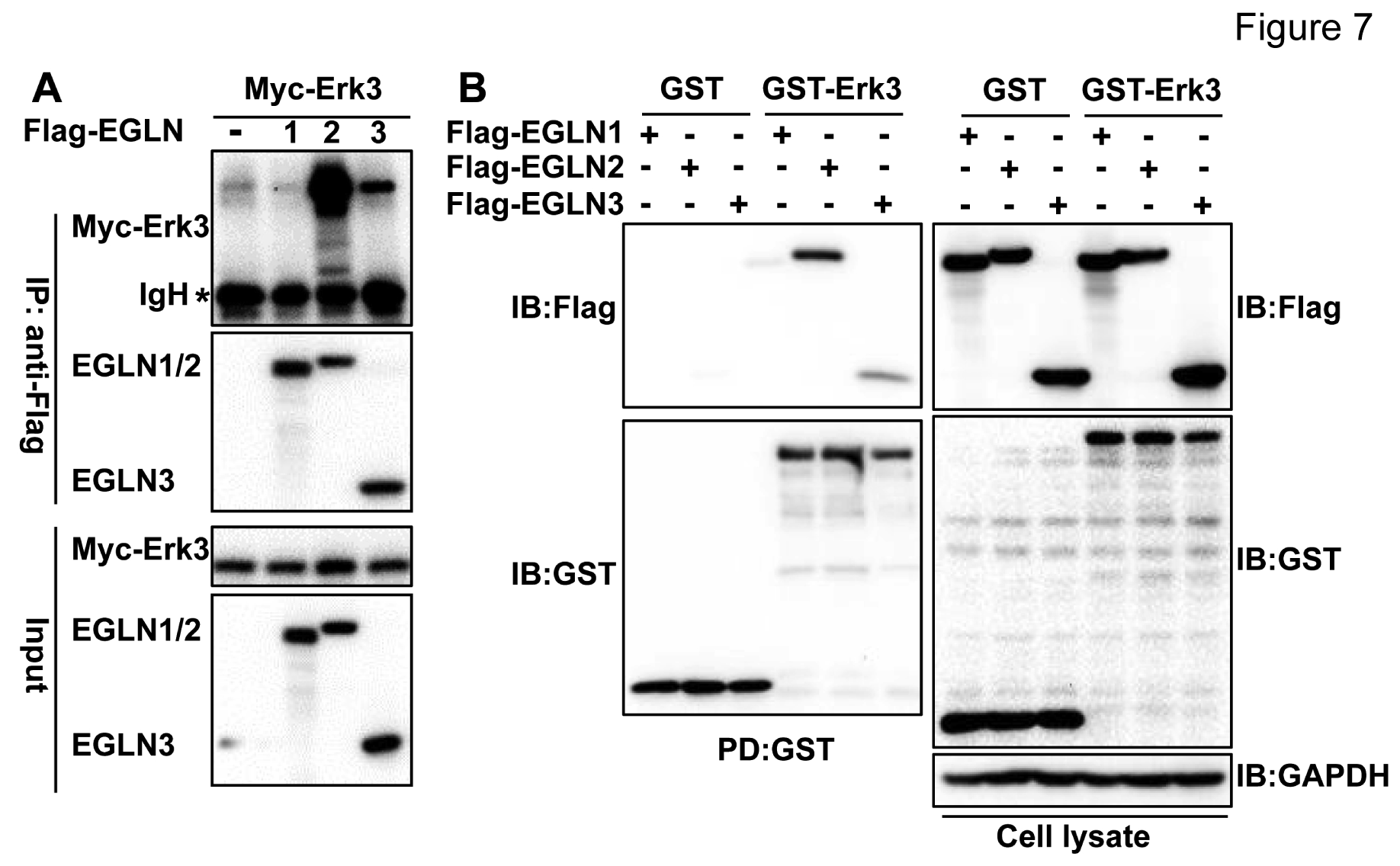

Supplement: Supplementary file 8 — Supplementary Fig. 7 EGLN3 selectively stabilized the Erk3 protein. [file 41388_2022_2203_MOESM8_ESM.tif]

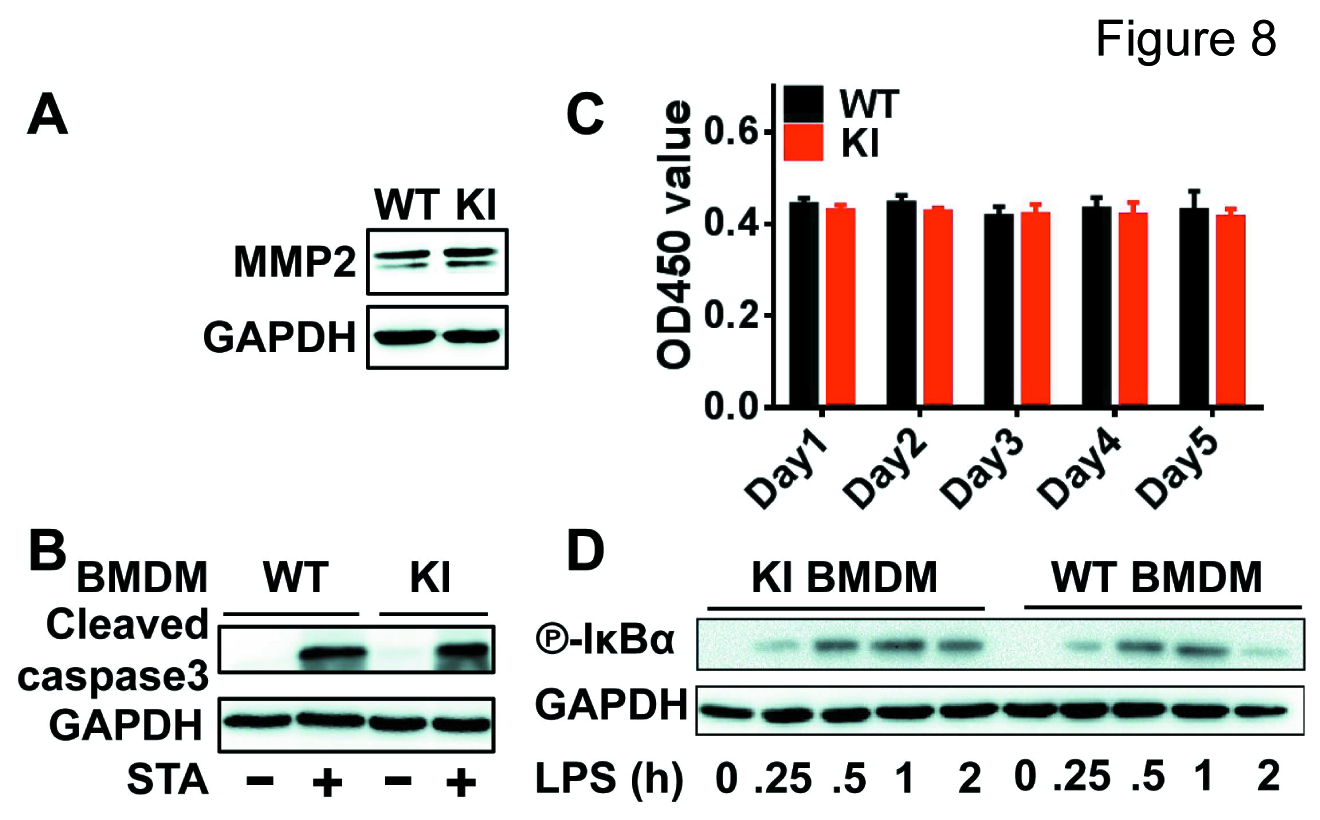

Supplement: Supplementary file 9 — Supplementary Fig. 8 The effects of EGLN3 inactivation on the properties of macrophages. [file 41388_2022_2203_MOESM9_ESM.tif]

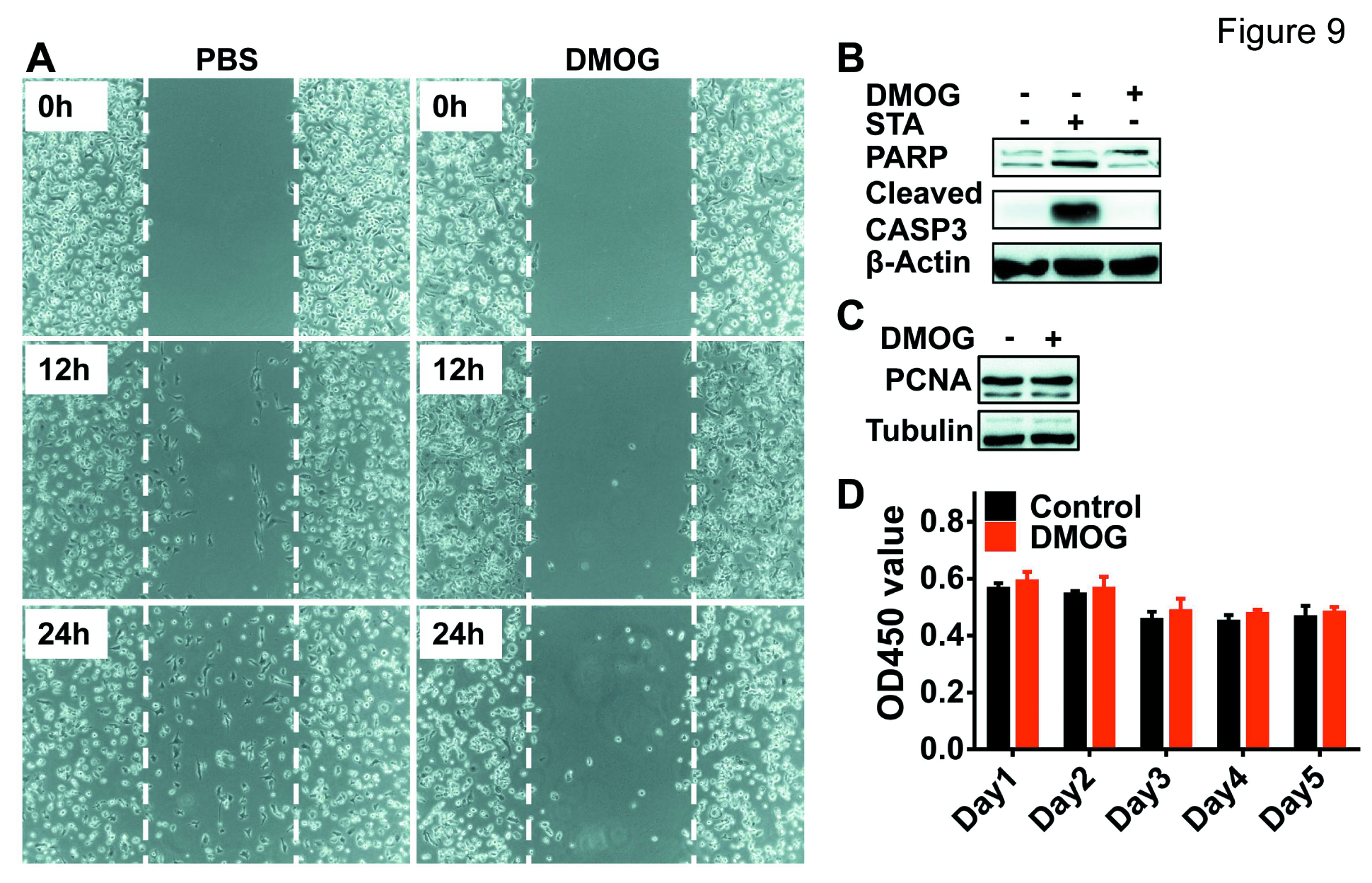

Supplement: Supplementary file 10 — Supplementary Fig. 9 The effects of DMOG on the properties of macrophages. [file 41388_2022_2203_MOESM10_ESM.tif]

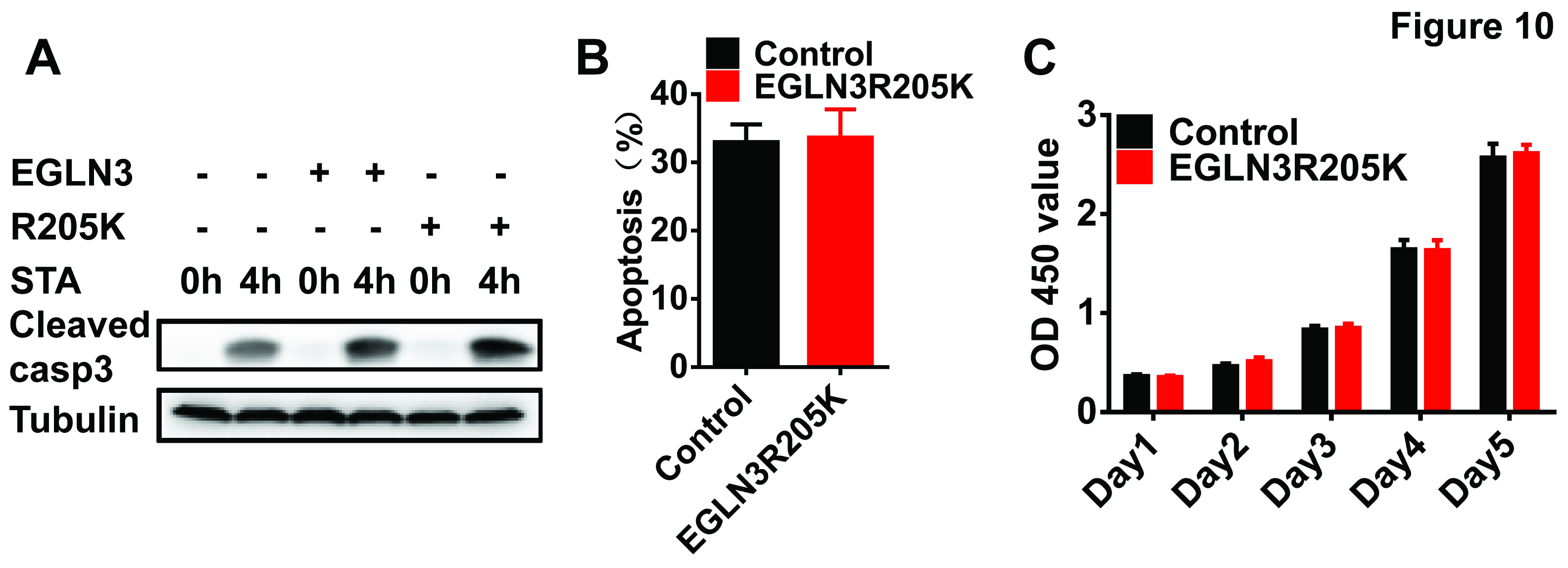

Supplement: Supplementary file 11 — Supplementary Fig. 10 Expression of hydroxylase-inactive EGLN3 had no effects on proliferation and apoptosis of LLC lung cancer cells. [file 41388_2022_2203_MOESM11_ESM.tif]
